# Nanomaterials for IoT Sensing Platforms and Point-of-Care Applications in South Korea

**DOI:** 10.3390/s22020610

**Published:** 2022-01-13

**Authors:** Seung-Ho Choi, Joon-Seok Lee, Won-Jun Choi, Jae-Woo Seo, Seon-Jin Choi

**Affiliations:** 1 Division of Materials of Science and Engineering, Hanyang University, 222 Wangsimni-ro, Seongdong-gu, Seoul 04763, Korea; tmdgh1222@hanyang.ac.kr (S.-H.C.); dlwnstjr5375@hanyang.ac.kr (J.-S.L.); dkrakdnanf@hanyang.ac.kr (W.-J.C.); wodn2859@hanyang.ac.kr (J.-W.S.); 2 Institute of Nano Science and Technology, Hanyang University, 222 Wangsimni-ro, Seongdong-gu, Seoul 04763, Korea

**Keywords:** nanostructure, IoT, POCT, gas sensor, ion sensor, biosensor

## Abstract

Herein, state-of-the-art research advances in South Korea regarding the development of chemical sensing materials and fully integrated Internet of Things (IoT) sensing platforms were comprehensively reviewed for verifying the applicability of such sensing systems in point-of-care testing (POCT). Various organic/inorganic nanomaterials were synthesized and characterized to understand their fundamental chemical sensing mechanisms upon exposure to target analytes. Moreover, the applicability of nanomaterials integrated with IoT-based signal transducers for the real-time and on-site analysis of chemical species was verified. In this review, we focused on the development of noble nanostructures and signal transduction techniques for use in IoT sensing platforms, and based on their applications, such systems were classified into gas sensors, ion sensors, and biosensors. A future perspective for the development of chemical sensors was discussed for application to next-generation POCT systems that facilitate rapid and multiplexed screening of various analytes.

## 1. Introduction

The development of high-performance chemical sensors has become increasingly important in recent years because of industrialization and the need to prevent widespread viral infections. Additionally, chemical sensors for biomarker detection in the human body are gaining considerable attention for the non-invasive diagnosis of diseases and the monitoring of health conditions in real time [1,2]. Various analytes such as gases, ions, and biocomponents (e.g., glucose, viruses, and bacteria) can be detected using chemical sensors, thereby making such sensors suitable for applications in environmental monitoring and healthcare. To detect trace amounts of analytes, various sensing parameters, particularly high sensitivity and selectivity, must be considered. Moreover, the rapid detection of target analytes is essential for preventing the spread of hazardous chemical species and detecting abnormal health states within a short time. Furthermore, the miniaturization of sensor platforms with low power consumption is necessary for portable on-site detection and point-of-care testing (POCT) [3,4,5].

**Figure 1:**
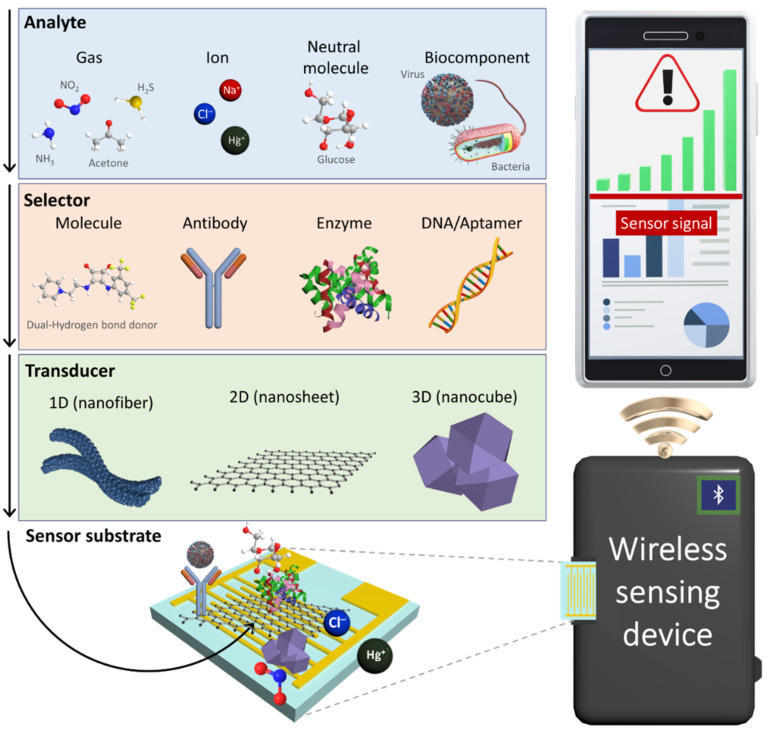
Schematic illustration of an IoT sensing platform comprising a chemical sensor, a wireless sensing device, and a smartphone for POCT application. Electrochemical sensors using nanomaterials consisting of selectors and transducers produce electrical signals upon chemical interactions with various analytes such gases, ions, neutral molecules, and biocomponents.

To develop next-generation chemical sensors, the development of novel sensing materials and their integration with sensing systems are desired (Figure 1). For the development of chemical sensing layers, several nanomaterials with structural engineering have been proposed to facilitate their large surface area and high porosity considering that the fundamental sensing mechanism is the result of surface chemical reactions [6,7,8,9]. For example, multidimensional nanostructures such as zero-dimensional (0D) nanoparticles [10], one-dimensional (1D) nanofibers [11], two-dimensional (2D) nanosheets [12,13], and three-dimensional (3D) nanocubes have been demonstrated to be effective sensing layers with high sensitivity [14,15]. In addition, attempts have been devoted toward the tuning of physical properties such as electrical conductivity and optical emission of nanomaterials by compositional and chemical reactivity modulations [16]. These nanomaterials serve as a transducer layer on a sensing substrate to produce distinguishable signal outputs owing to the changes in their electrical and optical properties. To enhance selectivity by inducing specific binding, various selectors (e.g., synthetic molecules [17], antibodies [18], enzymes [19], and DNAs/aptamers [20]) have been functionalized with nanomaterials [21]. Chemical interactions between analytes and selectors can be effectively transduced into electrical or optical signals through the transducer layer.

In sensing systems, sensing data are transmitted to a personal mobile device through wireless communication based on the Internet of Things (IoT) platform. Chemical sensors integrated with portable IoT devices have been employed for the real-time and on-site detection of target analytes in order to enable POCT applications [22,23]. For example, wearable sensor systems were fabricated by combining a transducer layer with a flexible substrate to quantitatively monitor chemical analytes in body fluids, and the measured sensing data were displayed on a smartphone [24,25,26,27]. The sensing systems assembled with nanomaterials can be further optimized for integration with IoT sensing platforms, depending on their applications and target analytes.

In this review, we discuss recent achievements in the development of chemical sensors in which novel nanomaterials are integrated with IoT sensor systems for POCT applications in South Korea. The synthesis methods, characterization, and sensing properties of nanomaterials are discussed in detail for their application toward the detection of various analytes such as gases, ions, neutral molecules, and biocomponents. Based on their application, these nanomaterials are classified into gas sensors, ion sensors, and biosensors. This comprehensive review reveals the current research state and provides future directions toward the development of chemical sensors comprising innovative sensing materials and systems for their application in the next-generation POCT platforms.

## 2. Gas Sensors

Nanomaterials integrated with IoT-based gas-sensing modules are gaining considerable attention for their use in monitoring hazardous environments, food freshness, and disease diagnosis [28,29]. For example, nitrogen dioxide (NO_2_) is a toxic gas emitted from automobiles and industrial plants that causes respiratory diseases under excess exposure [30]. In addition, highly sensitive gas sensors that detect various volatile organic compounds can be employed in diagnostic POCT applications [31]. For instance, the analysis of acetone concentration in exhaled breath can provide information about the metabolic state, such as body fat burning and diabetic symptoms [32,33].

Among the various types of gas sensors, chemiresistive gas sensors are suitable for integration with IoT sensing platforms because of their simple working principle, ease of fabrication, and low cost [34]. The performance of chemiresistive gas sensors depends on the microstructures of nanomaterials, considering that sensing signals are transduced by surface chemical reactions. To further improve the gas-sensing performance, microstructural and compositional modifications have been attempted using novel synthesis techniques [35]. In this section, we review the recent research progress in South Korea on the development of gas sensors using multidimensional nanocomposites, which can be integrated with IoT sensing platforms, and the use of such sensors in POCT applications. 

1D nanostructures have been employed as gas-sensing layers owing to their large surface area and porosity, which allows for a high sensitivity [36,37,38,39]. Moreover, 1D nanomaterials with controlled structures and morphologies have been developed as gas-sensing layers, such as nanorods (NRs) [40], nanowires (NWs) [41], nanofibers (NFs) [42], and nanotubes (NTs) [43]. 

**Figure 2:**
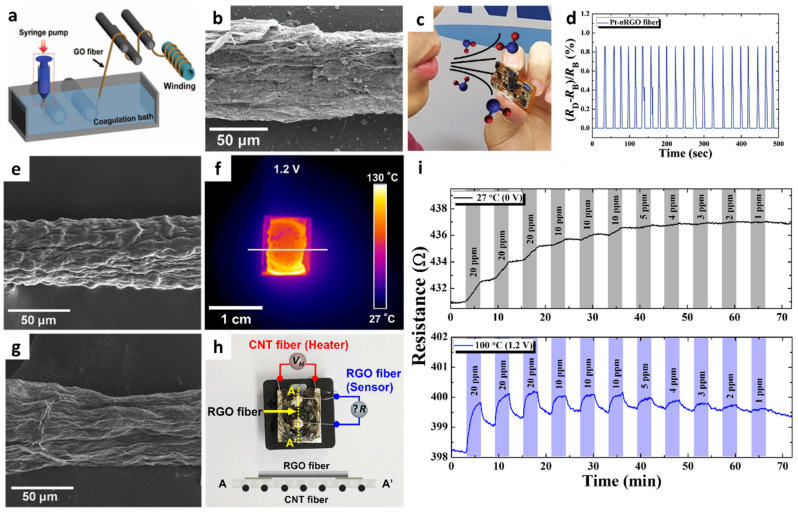
(**a**) Schematic illustration of the synthesis of a GO fiber via wet spinning. (**b**) SEM image of Pt-nRGO fiber after optothermal sintering upon IPL irradiation. (**c**) Exhaled breath injection to the Pt-nRGO fiber sensor integrated with a portable sensing module. (**d**) Real-time response transitions during breath humidity monitoring by using the sensing module. Reproduced with permission from Ref. [46] Copyright (2018), Wiley-VCH. (**e**) SEM image of the CNT fiber synthesized via wet spinning. (**f**) IR image of a CNT fiber-cPI film as a heater under an applied voltage of 1.2 V. (**g**) SEM image of an RGO fiber synthesized via wet-spinning. (**h**) Camera image of an all-carbon fiber-based sensor fabricated by the integration of an RGO fiber on a CNT fiber-cPI film with the schematic image of the cross-sectional structure. (**i**) Resistance transitions of an all-carbon fiber-based sensor at different operating temperatures (applied voltages). Reproduced with permission from Ref. [51] Copyright (2019), Elsevier.

1D graphene fibers have become a research focus considering that fibrous structures with high mechanical strength and tensile modulus can be integrated with wearable chemical sensors for the on-site detection of gas species [44,45]. A sensor using nitrogen-doped reduced graphene oxide (nRGO) fibers functionalized with Pt nanoparticles (NPs) was developed for application in wearable humidity sensors [46]. The nRGO fiber was produced by a wet-spinning process followed by heat-treatment in a reducing ambient. Continuously aligned graphene oxide (GO) fibers were obtained by lyotropic liquid crystals (LCs) property during the wet-spinning process [47]. Specifically, a 1.8 wt% GO solution was coagulated in a CaCl_2_ solution, and the aligned GO sheets were precipitated into a continuous fiber structure under ejection through a syringe nozzle (Figure 2a). GO fibers with a diameter range of 50–80 μm were obtained after washing and drying. Subsequently, the GO fibers were annealed at 900 °C in a reducing atmosphere (H_2_/N_2_, 5%/95%, *v*/*v*) to form nRGO fibers, resulting in nitrogen doping and removal of oxygen functional groups [48]. The X-ray photoelectron spectroscopy (XPS) analysis revealed that the RGO fiber was reduced by the formation of C–N bond and removal of oxygen functional groups, which resulted in the improved electrical conductivity of nRGO.

To functionalize Pt NPs on the nRGO fiber, a hollow protein cage (i.e., apoferritin) was used as a template. Apoferritin is composed of peptide subunits with an inner cavity diameter of 8 nm and overall size of 13 nm [49]. The protein cage can encapsulate Pt ions inside the protein cavity, and the subsequent reduction process results in metallic Pt NPs encapsulated by protein cages. Optothermal sintering upon intense pulse light (IPL) irradiation was performed using a xenon flash lamp to remove protein templates and form Pt NPs on the nRGO fiber (Pt-nRGO) (Figure 2b). 

A colorless polyimide (cPI) film was prepared as a substrate for the Pt-nRGO humidity sensor. As a precursor solution for cPI film, a polyamic acid (PAA) solution was first prepared by mixing 4,4-(hexafluoroisopropylidene)diphthalic anhydride and 3,3-diaminodiphenyl sulfone in *N,N*-dimethylacetamide. Next, the PAA solution was coated on a glass substrate by the screen-printing method followed by imidization at 100 °C, 200 °C, and 230 °C for 1 h at each temperature to form a cPI film. After patterning the sensing electrodes on the cPI film, the Pt-nRGO fiber was electrically connected between the two electrodes to measure resistance changes.

Humidity sensing properties were investigated by measuring the resistance transitions of Pt-nRGO fibers on a flexible cPI substrate at different relative humidity (RH) levels. The response of the sensors was calculated as ((R_H_–R_D_)/R_D_ (%)), where R_H_ and R_D_ are the resistance upon exposure toward humid air (i.e., 6.1–99.9% RH) and baseline dry air (i.e., 2.6% RH), respectively. The pristine nRGO fiber exhibited responses of 0.27% at 6.1% RH and 3.53% at 66.4% RH. The Pt-nRGO fiber exhibited improved humidity responses of 0.32% at 6.1% RH and 4.51% at 66.4% RH. The improved humidity sensing properties of Pt-nRGO were mainly attributed to the uniform functionalization of Pt with a particle size of ~2 nm through catalytic water dissociation [50]. The Pt-nRGO fiber was integrated with a portable sensing module to demonstrate its applicability for the real-time and on-site detection of humidity changes under direct exposure to human exhaled breath (Figure 2c). Consistent response transitions of 0.86% were observed after repetitive injections of exhaled breath to the sensor for 2 s (Figure 2d). The stable sensing property of Pt-nRGO was mainly attributed to the favorable adsorption of water molecules owing to the presence of numerous defect sites at the domain boundary of nRGO and the catalytic effect of Pt NPs by dissociation of water molecules. Because of its unique fibrous structure and outstanding humidity sensing properties, Pt-nRGO can be applied for the detection of biomarkers in exhaled breath.

Another promising application of 1D graphene fibers is environmental monitoring through the on-site detection of toxic gases such as NO_2_. However, graphene-based sensing layers suffer from incomplete recovery and drift in baseline resistance after exposure to NO_2_ as a result of the irreversible recovery process. To achieve reversible NO_2_ sensing, a flexible heating substrate was prepared by embedding carbon nanotube (CNT) fibers in a cPI film and integrating it with a graphene fiber [51]. Continuous CNT fibers were synthesized by the wet-spinning process, in which purified CNT powder dispersed in chlorosulfonic acid was ejected through a syringe nozzle in a coagulation bath. The CNT fiber exhibited a 1D structure with preferentially aligned CNTs along the axial direction (Figure 2e). A CNT fiber-embedded cPI (CNT fiber-cPI) film was fabricated by dispersing CNT fiber networks in PAA followed by the imidization process. The heating property of a CNT fiber-cPI heater was characterized by applying a voltage of 0–1.2 V to the CNT fiber-cPI film. Voltage-dependent current transitions were observed with an increase in the film temperature. The infrared image clearly shows the heating property of the CNT fiber-cPI heater with an operating temperature of 90.5 °C at an applied voltage of 1.2 V (Figure 2f).

For the sensing layer, RGO fibers were prepared by the wet-spinning process followed by thermal reduction. GO fibers were first produced by the wet-spinning process, similar to the synthesis of CNT fibers. Subsequently, heat-treatment was performed at 900 °C in a reducing atmosphere (H_2_/N_2,_ 4%/96%) for 2 h to form RGO fibers. The continuous fibrous structure of RGO was maintained with RGO sheets aligned on the surface (Figure 2g). The RGO fiber was deposited on a CNT fiber-cPI film to produce an all-carbon fiber-based sensor (Figure 2h). 

The sensing property of the all-carbon fiber-based sensor was investigated toward NO_2_ in the concentration range of 1–20 ppm under different operating temperatures controlled by the CNT fiber-cPI heater. (Figure 2i). Although the RGO fibers showed a noticeable response to 20 ppm NO_2_ at room temperature, the recovery was negligible, resulting in a severe drift in the sensor signal. In addition, the resistance changes of the RGO fibers were negligible at concentrations below 5 ppm at room temperature. On the other hand, further improved response and recovery properties were achieved when the operating temperature was increased to 100 °C (1.2 V). The theoretical detection limit was calculated to be 814 ppb at 100 °C, implying that the all-carbon fiber-based sensor can potentially detect NO_2_ at sub-ppm levels.

To quantitatively analyze the reversible NO_2_ reaction and recovery processes, adsorption and desorption kinetics were evaluated by calculating the reaction rate constants, i.e., the desorption rate constant (k_des_) and adsorption rate constant (k_ads_), based on the following equations [51,52,53]:

(1) $$S\left(t\right){=S}_{0}\exp\left[-k_{\mathrm{des}}t\right]$$

(2) $$S\left(t\right){=S}_{\max}\frac{C_{a}K}{{1+C}_{a}K}\left(1-\exp\left[-\frac{{1+C}_{a}K}{K}k_{\mathrm{ads}}t\right]\right)$$

where S_0_ is the response when the analyte gas is removed, S_max_ is the maximum response toward the analyte gas, and C_a_ is the concentration of the analyte gas. A relatively low adsorption rate constant (k_ads_ = 2.48 × 10^−2^ ppm^−1^ s^−1^) was obtained at room temperature. Moreover, the negative desorption rate constant (k_des_ = −1.34 × 10^−3^ s^−1^) indicates negligible recovery upon exposure to air at room temperature. On the other hand, a 2.17-fold increase in response kinetics (k_ads_ = 5.37 × 10^−2^ ppm^−1^ s^−1^) was achieved by increasing the operating temperature to 100 °C. In particular, substantially improved recovery kinetics were achieved with a 9.22-fold enhancement in the desorption rate constant (k_des_ = 8.85 × 10^−3^ s^−1^) at 100 °C. This work paves the way for the development of next-generation chemical sensors using unique carbonaceous fibers as a sensing layer, as well as a heating element for the detection of toxic chemicals with improved reversibility.

**Figure 3:**
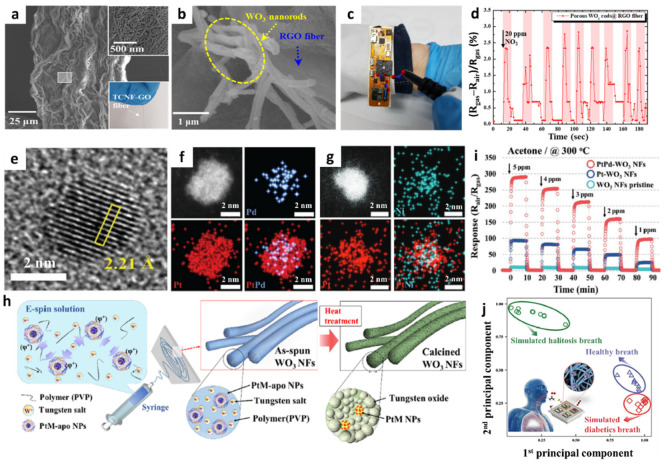
SEM images of (**a**) TCNF-GO and (**b**) porous WO_3_ NRs-RGO fibers. (**c**) Digital image of a portable sensing module loaded with porous WO_3_ NRs-RGO fibers. (**d**) Real-time NO_2_ sensing property using a portable sensing module. Reproduced with permission from Ref. [57] Copyright (2019), American Chemical Society. (**e**) High-resolution TEM image of PtPd-apo NPs. EDS elemental mapping images of (**f**) PtPd-apo NPs and (**g**) PtNi-apo NPs. (**h**) Schematic illustration of the electrospinning process for the synthesis of mesoporous WO_3_ NFs functionalized with PtM NPs by the apoferritin-encapsulating method. (**i**) Sensing properties of pristine WO_3_ NFs, Pt-WO_3_ NFs, and PtPd-WO_3_ NFs toward acetone in the concentration range of 1–5 ppm at 300 °C. (**j**) Pattern recognition of exhaled breath using sensor arrays, demonstrating their applicability for non-invasive POCT for diabetes and halitosis diagnosis. Reproduced with permission from Ref. [61] Copyright (2017), Wiley-VCH.

The use of multi-compositional 1D structures is an effective way to improve the gas-sensing performance [54]. Incorporating heterogeneous sensing materials that combine metal oxides on a conductive carbon framework can facilitate the development of high-performance gas sensors through the activation of the physical/chemical adsorption properties of gas species [55,56]. Jang et al. fabricated porous RGO fibers functionalized with WO_3_ NRs by employing wet-spinning and solution-based self-assembly processes [57]. The formation of abundant pore sites on the GO fiber is advantageous for improving the gas response through the promotion of gas penetration and the acceleration of the surface reaction. Thus, tunicate cellulose nanofibers (TCNFs) were prepared by forming porous GO fibers [58]. During the wet-spinning process, TCNF and GO were wound into a fibrous structure (TCNF-GO) by exploiting the LC properties in an aqueous solution. As a result, a TCNF-GO fiber with a unique wrinkled surface morphology and well-distributed mesopores was obtained (Figure 3a). To form WO_3_ NRs on the porous RGO fiber (porous WO_3_ NRs-RGO), the solution-based self-assembly process was performed by inducing the adsorption of a tungsten precursor on a hydrophilic TCNF, resulting in the uniform distribution of the tungsten precursor on the TCNF-GO fiber [59]. After heat-treatment in an argon atmosphere at 700 °C, WO_3_ NRs were grown on RGO fibers with a mean width of 197 nm (Figure 3b). 

Gas-sensing characterization of the WO_3_ NRs-RGO fiber was performed at 100 °C toward 5 ppm NO_2_, which revealed a high response (|R_gas_–R_air_|/R_air_ × 100 (%)) of 9.67%. In addition, notable selectivity toward NO_2_ was confirmed with minor responses (<2.45%) toward other interfering gases such as ethanol (C_2_H_5_OH), acetone (C_3_H_6_O), toluene (C_7_H_8_), hydrogen sulfide (H_2_S), and nitrogen monoxide (NO). The porous WO_3_ NRs-RGO fiber was integrated with a wristband-type sensing module to demonstrate its applicability in wearable sensors (Figure 3c). After the injection of 20 ppm NO_2_ for 10 cycles, consistent and reversible NO_2_ sensing properties were obtained with a response range of 2.25–2.75% at room temperature (Figure 3d). The improved NO_2_ sensing performance of the porous WO_3_ NRs-RGO fiber was mainly attributed to the heterojunction effect between the WO_3_ NRs and RGO fibers facilitating an effective surface reaction and the charge transduction properties [60].

Sacrificial templates can be utilized to form porous nanostructures and to transport catalytic NPs to the sensing layer. In particular, well-dispersed catalytic NPs with multiple compositions can be synthesized by encapsulating them in a sacrificial template. Kim et al. proposed a new approach to synthesize Pt-based bimetallic catalysts (PtM, M = Pd, Ru, and Ni) on mesoporous WO_3_ NFs by employing the encapsulating route by using apoferritin protein nanocages [61]. Highly dispersed bimetallic PtM NPs were obtained by the reduction of both Pt and metal (Pd, Ru, Ni) ions in the apoferritin hollow nanocage (PtM-apo), resulting in average particle size of less than 3 nm. The high-resolution TEM image of PtPd NPs showed an interplanar distance of 2.21 Å, implying the formation of an intermetallic PtPd compound (Figure 3e). Elemental distribution of PtM NPs was confirmed by energy-dispersive X-ray spectroscopy (EDS) mapping analysis; overlapping images for both Pt and Pd elements were observed for PtPd-apo (Figure 3f). On the other hand, PtNi-apo showed a scattered elemental distribution as a result of the difference in the reduction rate between Pt and Ni, leading to the increased size of PtNi-apo compared to that of PtPd-apo (Figure 3g).

The electrospinning process was performed to form WO_3_ NFs functionalized with PtM NPs (PtM-WO_3_ NFs) (Figure 3h). An electrospinning solution was prepared by dissolving tungsten precursor ((NH_4_)_6_H_2_W_12_O_40_·xH_2_O) and polyvinylpyrrolidone (PVP) in deionized (DI) water followed by the homogeneous dispersion of PtM-apo NPs to obtain the nanofibrous composite 1D structure of W precursor/PVP/PtM-apo. After heat-treatment at 600 °C, porous WO_3_ NFs functionalized with catalytic PtM NPs were obtained as a result of the decomposition of protein shells and the transfer of PtM NPs onto WO_3_ NFs. 

Gas-sensing properties of the pristine WO_3_ NFs, Pt-WO_3_ NFs, and PtPd-WO_3_ NFs toward acetone were evaluated in the concentration range of 1–5 ppm at 300 °C. The gas response of PtPd-WO_3_ NFs (R_air_/R_gas_ = 97.5) was substantially improved as compared to those of the pristine WO_3_ NFs (R_air_/R_gas_ = 4.3) and Pt-WO_3_ NFs (R_air_/R_gas_ = 24.9) toward 1 ppm acetone at 300 °C (Figure 3i). In addition, the PtNi-WO_3_ NFs exhibited a drastically improved gas response of 340 toward 1 ppm H_2_S at 300 °C. The drastic improvement in the H_2_S sensing performance was mainly attributed to the dual catalytic effect of PtNi-WO_3_ NFs induced by the phase separation between Pt and NiO after the calcination process. The PtPd-WO, PtRu-WO, and PtNi-WO_3_ NFs were combined as a sensor array to analyze cross-selectivity and exhaled breath patterns. Simulated exhaled breath composed of healthy human breath containing 1 ppm of acetone and H_2_S was exposed to a sensor array to demonstrate its applicability for the diagnosis of diabetes and halitosis, respectively. As a result, separated patterns depending on the exhaled breath composition were obtained by principal component analysis (PCA), which verified the applicability of PtM-WO_3_ NFs for non-invasive diagnosis through the detection of exhaled breath biomarkers (Figure 3j).

**Figure 4:**
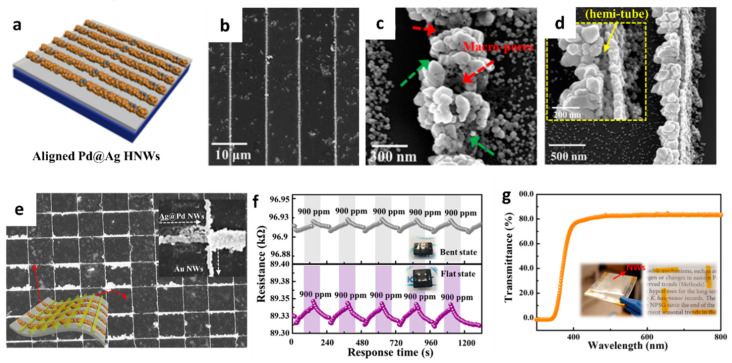
(**a**) Schematic illustration of Pd@Ag HNWs on a cPI film attached to a glass substrate. SEM images of the (**b**,**c**) Pd@Ag HNWs after the GRR for 17 h, (**d**) overturned Pd@Ag HNWs with a magnified image in the inset, and (**e**) grid-type NWs composed of Pd@Ag HNWs and Au NWs. (**f**) Resistance transitions of the heterogeneous Pd@Ag HNWs on a flexible cPI substrate toward 900 ppm H_2_ in flat and bent states. (**g**) The transmittance of the heterogeneous NWs composed of Pd@Ag HNWs and Au NWs. Reproduced with permission from Ref. [65] Copyright (2017), American Chemical Society.

The formation of porous and hollow structures can offer high gas permeability and accelerate the surface reaction [62,63,64]. A new synthesis strategy was proposed by aligning 1D nanostructures on a flexible substrate [65]. Hollow nanowires (HNWs) of Pd–Ag composite were synthesized by lithographically patterned nanowire electrodeposition (LPNE) and the subsequent galvanic replacement reaction (GRR) on a flexible cPI film for a reversible hydrogen (H_2_) reaction. The aligned Ag NWs were formed on the cPI film attached to a glass substrate by using the LPNE method (Figure 4a). Specifically, the Ni film was deposited by thermal evaporation as an etch mask during the patterning process followed by spin-coating of the photoresist to form a line pattern. Next, the sample was immersed in an etchant solution to form a line pattern of Ni by undercut etching below the photoresist while forming trenches. Electrodeposition was performed to form Ag NWs on the surface of Ni using the trenches as a template followed by the removal of the photoresist and Ni. To induce the transformation of Ag NWs to Pd-functionalized Ag HNWs (Pd@Ag HNWs), the GRR was performed by immersing the electrodeposited Ag NWs into the aqueous Pd precursor for a certain duration. SEM analysis revealed the optimum GRR time, wherein well-aligned Pd@Ag HNWs were obtained after 17 h (Figure 4b). A porous structure was formed on the surface of the Pd@Ag HNWs during the GRR (Figure 4c). In addition, the Pd@Ag HNWs with the continuous hemitubular structure were obtained owing to the standard reduction potential difference between Pd and Ag, resulting in the dissolution of Ag NWs and the subsequent growth of metallic Pd (Figure 4d). XPS revealed the formation of the Pd–Ag alloy during the replacement reaction.

To investigate the H_2_ sensing property, sensing electrodes comprising Au NWs were electrodeposited on a cPI film across the Pd@Ag HNWs (Figure 4e). The response (∆R/R_0_ × 100 (%)) of the Pd@Ag HNWs on the cPI film was 0.89 ± 0.01% toward 900 ppm H_2_ at room temperature in the flat state. A slightly decreased H_2_ response of 0.65 ± 0.03% at 900 ppm was observed when the cPI film was bent at an angle (*θ_b_*) of 30°. Nevertheless, reliable and reversible sensing properties were achieved in both bent and flat states upon multiple cyclic exposures to 900 ppm H_2_ (Figure 4f). The enhanced H_2_ sensing properties of the Pd@Ag HNWs were mainly attributed to the catalytic effect of Pd on the surface of the hollow structure induced by the formation of the PdH_x_ phase [66]. The Pd@Ag HNWs on the cPI film was formed by the unique fabrication technique combining electrodeposition and the GRR, which can be applied to develop flexible and transparent H_2_ sensors (Figure 4g).

For decades, 2D materials have been intensively studied for application in chemical sensors owing to the atomically thin layered geometry, adjustable electrical properties, and presence of abundant active edge sites [13,67,68]. Various 2D materials exhibit intriguing gas-sensing properties because of their large surface area and high surface-to-volume ratio. Moreover, the mechanical flexibility of 2D materials is advantageous for the fabrication of flexible and wearable gas sensors [69]. To date, numerous 2D materials such as graphene, transition metal dichalcogenides (TMDs), metal oxides, and black phosphorus have been developed for application as gas-sensing layers [70,71,72].

**Figure 5:**
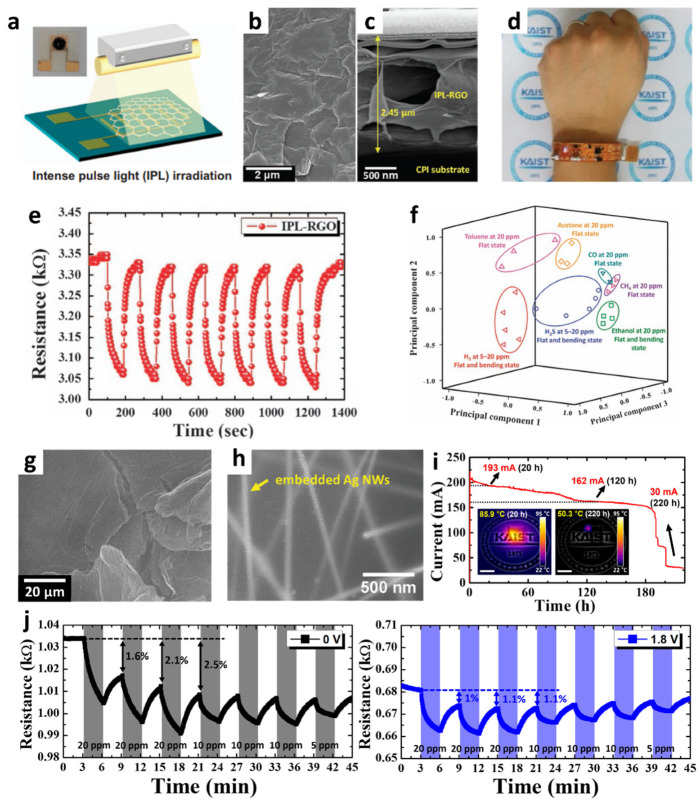
(**a**) Schematic illustration of IPL irradiation of GO sheets coated on a cPI film. SEM images of IPL-RGO sheets: (**b**) planar and (**c**) cross-sectional views. (**d**) Camera image of a wearable wristband-type sensor module integrated with the IPL-RGO sensor. (**e**) Dynamic resistance transitions toward 20 ppm H_2_S at room temperature. (**f**) PCA using the IPL-RGO sensor for pattern recognition of H_2_, H_2_S, ethanol, acetone, toluene, carbon monoxide, and methane at 5–20 ppm. Reproduced with permission from Ref. [75] Copyright (2016), Nature Publishing Group. SEM images of the (**g**) ORGO sheets on an Ag NW-cPI film and (**h**) Ag NWs embedded on a cPI film. (**i**) Current transition property of the Ag NW-cPI film during continuous operation at a constant applied voltage of 2 V with infrared images for temperature measurement. (**j**) Resistance transition characteristic of the ORGO layers on an Ag NW-cPI film at 25 and 71.7 °C controlled by the applied voltage. Reproduced with permission from Ref. [52] Copyright (2016), Wiley-VCH.

After the invention of isolated graphene sheets by mechanical exfoliation, their derivatives, such as GO and RGO, were investigated for their applicability as efficient sensing materials with enhanced gas-sensing properties [73,74]. Moreover, a wearable chemical sensor was developed for the detection of H_2_S by assembling RGO sheets on a cPI film [75]. A flexible cPI film was synthesized by the solution screen-printing method and the subsequent imidization process for use as a flexible substrate. To measure the resistance changes of the sensing layer, interdigitated electrodes with 200 µm spacing between the electrodes were patterned on the cPI film. The dispersed GO in a DI solution (2 mg mL^−1^) was drop-coated onto the cPI substrate. Subsequently, the GO sheets were reduced upon ultrafast optical irradiation by using IPL to form RGO (IPL-RGO) (Figure 5a). IPL irradiation is a facile method for generating heat in milliseconds without damaging the substrate [76]. After IPL irradiation, the electrical conductivity of the RGO sheets significantly increased as compared to that of the GO sheets. XPS and Raman spectra confirmed the reduction of GO by eliminating the oxygen functional groups on the surface. The morphological transition of the IPL-RGO sheets was investigated through SEM (Figure 5b–c). IPL-RGO exhibited a rough surface morphology and had numerous open pores, which effectively facilitated gas diffusion.

The gas-sensing performance of the IPL-RGO sheet sensor was investigated by injecting H_2_S, ethanol, and H_2_ in the flat and bent states. The IPL-RGO sensor showed characteristic resistance transitions during cyclic exposure to H_2_S, whereas the resistance changes were negligible for the pristine GO sensor. The response ((R_air_–R_gas_)/R_air_ (%)) of the IPL-RGO sensor was 0.238% and 0.224% toward H_2_S at 20 ppm in the flat and bent states, respectively. Furthermore, the IPL-RGO sensor was integrated with a wristband-type sensing module, and the sensing data were transmitted to a smartphone (Figure 5d). The results revealed that consistent and stable resistance transitions were observed after repeated exposure to H_2_S (Figure 5e). PCA was performed to visualize the classification of various analyte gases in the concentration range of 5–20 ppm when the IPL-RGO sensor was in the flat and bent states. All regions of the individual analytes were separated, demonstrating the classification of various analyte gases such as H_2_S, toluene, H_2_, and acetone using the IPL-RGO sensor (Figure 5f).

For the detection of NO_2_, RGO is frequently adopted as a sensing layer because of its high binding energy to NO_2_ at room temperature [77]. However, the slow recovery process and the low desorption rate of graphene sheets are critical challenges that limit the achievement of reversible NO_2_ sensing properties. To address these issues, a reversible gas-sensing system was developed by integrating optically reduced graphene oxide (ORGO) sheets on Ag NW-embedded cPI (Ag NW-cPI) heating film [52]. As a sensing layer, GO sheets were coated on the Ag NW-cPI heating substrate by using the drop-coating method. Subsequently, IPL irradiation was performed to convert GO to ORGO through optothermal energy. After IPL irradiation, multilayered 2D ORGO sheets were maintained with surface cracks, which was mainly attributed to the desorption of oxygen functional groups in the form of CO_2_ during the optothermal reduction process (Figure 5g).

To fabricate the Ag NW-cPI film, the Ag NWs were filtrated on a 0.2-μm pore nylon membrane and transferred to a glass substrate using a pressing machine. The PAA solution was coated on the Ag NW-transferred glass substrate, which was then imidized at elevated temperatures. Subsequently, the Ag NW-cPI film was detached from the glass substrate by immersion in DI water. As a result, a highly conductive and flexible heating substrate was obtained by partially embedding the Ag NW networks in the cPI film (Figure 5h). The long-term stability of the Ag NW-cPI heater was investigated by monitoring the temperature and current changes for 220 h at a constant voltage of 2 V (Figure 5i). The current levels slowly decreased over 120 h as a result of the regional breakdown of the Ag NW networks. After 180 h of operation, the temperature suddenly decreased to 50.3 °C, indicating substantial disconnections in the Ag NW networks.

The reversible NO_2_ sensing characteristics of the ORGO sheets were investigated by controlling the operating temperatures of an Ag NW-cPI heater (Figure 5j). There was a significant drift in the baseline resistance owing to the irreversibility of ORGO toward the reaction with NO_2_. Relatively large deviations (1.6–2.5%) from the initial baseline resistance were observed after exposure to 20 ppm NO_2_ at room temperature. In contrast, significantly reduced deviations of less than 1.1% were achieved at 71.7 °C when a voltage of 1.8 V was applied to the Ag NW-cPI film. Reaction rate constants were calculated for the ORGO sensor at different operating temperatures, i.e., 25 °C (0 V) and 71.7 °C (1.8 V). When the operating temperature was increased from 25 °C to 71.7 °C, a 1.3-fold increase in the adsorption rate constant (k_ads_) from 4.649 × 10^−3^ ppm^−1^ s^−1^ to 6.201 × 10^−3^ ppm^−1^ s^−1^ was observed. Similarly, a 1.7-fold increase in the desorption rate constant (k_des_) from 4.579 × 10^−3^ s^−1^ at 25 °C to 7.731 × 10^−3^ s^−1^ at 71.7 °C was obtained. This result indicates that both the reaction and recovery processes were accelerated when the flexible Ag NW-cPI heating film was used, thereby demonstrating the applicability of ORGO sheets for reversible NO_2_ detection.

**Figure 6:**
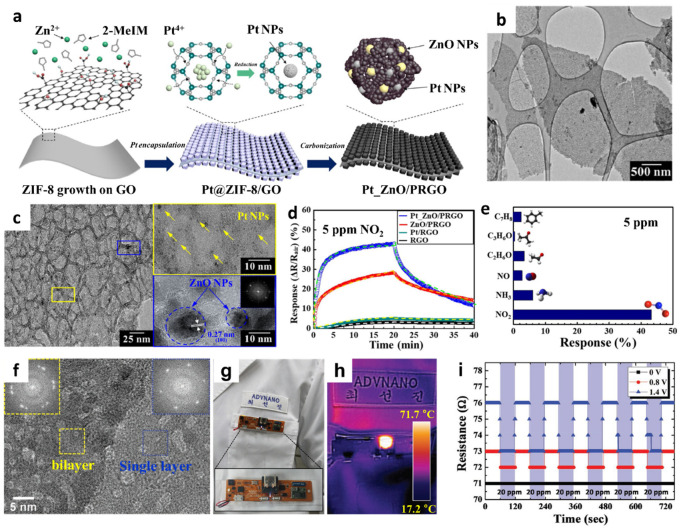
(**a**) Schematic illustration of the synthesis of PRGO functionalized with Pt and ZnO NPs (Pt_ZnO/PRGO) driven by MOF templates and subsequent pyrolysis. (**b**) TEM image and (**c**) high-resolution TEM image of Pt_ZnO/PRGO with Pt NPs (yellow box) and ZnO NPs (blue box). (**d**) Dynamic response transitions of pristine RGO, Pt/RGO, ZnO/PRGO, and Pt_ZnO/PRGO toward 5 ppm NO_2_ at room temperature. (**e**) Selective NO_2_ sensing property of Pt_ZnO/PRGO against interfering gases at 5 ppm. Reproduced with permission from Ref. [80] Copyright (2021), Elsevier. (**f**) TEM image of porous Ru oxide NSs with FFT diffraction patterns in the inset. (**g**) Camera image of a patch-type sensor module attached to a lab coat with the porous Ru oxide NS assembled on an Ag NW-cPI film. (**h**) Infrared camera image of the sensor module during operation at an elevated temperature. (**i**) Resistance transitions of Ru oxide NSs toward 20 ppm of NO_2_ under different applied voltages to the Ag NW-cPI heating film. Reproduced with permission from Ref. [82] Copyright (2017), Wiley-VCH.

To further enhance the gas-sensing performance, a compositional modification was proposed by employing a sacrificial templating route. Sacrificial templates can form a porous nanostructure in the sensing layer, thereby accelerating gas diffusion through the pores [78,79]. Recently, metal-organic framework (MOF)-driven 2D nanostructures have been developed on flexible substrates to form heterogeneous graphene-based sensing layers [80]. Porous reduced GO was functionalized with Pt and ZnO NPs (Pt_ZnO/PRGO) using MOF templates followed by pyrolysis (Figure 6a). The solution-phase synthesis method was adopted to grow ZIF-8 on GO (ZIF-8/GO) by combining ZIF-8 precursors with GO dispersion. To functionalize the Pt NPs, precursors comprising H_2_PtCl_6_·xH_2_O and PVP were dissolved in a ZIF-8/GO suspension. Subsequently, an aqueous NaBH_4_ solution was added to form Pt NPs via a reduction in the ZIF-8/GO suspension (Pt_ZIF-8/GO). Finally, calcination was conducted at 650 °C in an N_2_ ambient environment for 3 h to obtain Pt_ZnO/PRGO. The microstructure of Pt_ZnO/PRGO confirmed the layered structure of PRGO covered by ZnO polyhedrons (Figure 6b). During the calcination process, GO was transformed to PRGO and ZIF-8 was converted to hollow ZnO nanocages (Figure 6c). Well-dispersed Pt NPs (yellow box in Figure 6c) and ZnO NPs (blue box in Figure 6c) were confirmed through high-resolution TEM. The chemical composition of Pt_ZnO/PRGO was studied by XPS, wherein the characteristic peaks of Zn and Pt revealed the formation of ZnO and metallic Pt NPs, respectively.

The chemical sensing properties of NO_2_ at room temperature were determined by calculating the normalized response, i.e., (R_air_–R_gas_)/R_air_ × 100 (%). The pristine RGO, Pt/RGO, ZnO/PRGO, and Pt_ZnO/PRGO were exposed to 5 ppm of NO_2_ to investigate the effect of heterogeneous sensitization on gas response property. The Pt_ZnO/PRGO sensor exhibited the highest response (43.28%) than the other samples (Figure 6d). In addition, high NO_2_ selectivity was achieved with the Pt_ZnO/PRGO sensor with minor responses toward interfering gas analytes such as toluene, acetone, ethanol, NO, and ammonia (NH_3_) at 5 ppm (Figure 6e). The sensitive and selective NO_2_-sensing properties of Pt_ZnO/PRGO were mainly attributed to the dual-sensitization of MOF-templated Pt catalysts and effective charge transfer between ZnO and PRGO. To demonstrate its potential application in wearable sensors, Pt_ZnO/PRGO was coated on a flexible cPI film, and its sensing properties were determined under mechanical deformation. The Pt_ZnO/PRGO sensor exhibited reversible and consistent resistance transitions after 450 bending cycles at a bending angle of 90°.

Various 2D layers can be fabricated on flexible substrates, facilitating their potential applications in wearable gas sensors. Further optimization of the gas-sensing properties is possible by tailoring the surface morphology and porosity [81]. For example, atomically thin porous 2D Ru oxide nanosheets (NSs) were developed on a flexible heating substrate and integrated with a wearable patch-type NO_2_ sensing module [82]. The Ru oxide NSs were synthesized by the liquid-phase exfoliation of layered sodium ruthenate by the intercalation of Na^+^ with H^+^ in a hydrochloric acid (HCl) solution and then replaced with tetrabutylammonium ions [12,83].

To control the operating temperature of the sensing layer, an Ag NW-cPI film was prepared as a flexible heating substrate [84]. The electrodes were patterned on an Ag NW-cPI substrate to detect the resistance changes in the sensing layer, followed by the drop-coating of Ru oxide NSs on the substrate. Finally, optical irradiation was performed using IPL to form nanoscale pores on the Ru oxide NSs. A transmission electron microscopy (TEM) confirmed the presence of numerous pores with diameters of less than 5 nm (Figure 6f). Moreover, fast Fourier transform (FFT) diffraction patterns confirmed single and bilayer Ru oxide NSs even after IPL irradiation, which was mainly attributed to the ultrafast IPL irradiation of optothermal energy (inset of Figure 6f). The surface chemical composition of the porous Ru oxide was investigated by XPS, which revealed the formation of fully oxidized and dehydrated RuO_2_ NSs after IPL irradiation.

To investigate the temperature dependent NO_2_ sensing characteristics of porous Ru oxide NSs, their resistance transitions were analyzed upon the application of a voltage to the flexible heater in flat and bent states. The responses (∆R/R_0_ (%))of the porous Ru oxide NSs were 1.124% and 1.116% toward 20 ppm NO_2_ in the flat and bent states, respectively, at an applied heating voltage of 1.4 V (80.3 °C). The porous Ru oxide NSs were integrated with a patch-type sensing module to demonstrate their applicability in wearable NO_2_ sensors (Figure 6g). The wireless sensor module was attached to clothing, and a heating voltage of 1.4 V was applied to maintain the operating temperature at ~70 °C (Figure 6h). The resistance transitions of porous Ru oxide NSs were observed at different applied voltages under cyclic exposure to 20 ppm NO_2_ (Figure 6i). The results revealed that improved adsorption and desorption kinetics were achieved on the porous Ru oxide NSs at elevated temperatures by applying a voltage to the Ag NW-cPI heating film.

**Figure 7:**
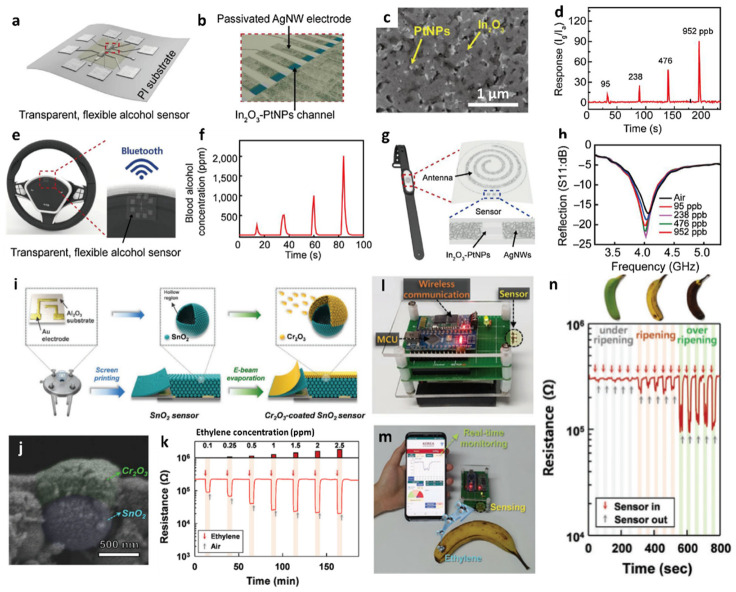
Schematic illustrations of (**a**) flexible and transparent alcohol sensor and (**b**) In_2_O_3_–Pt NP hybrid channel layer patterned with Ag NW electrodes. (**c**) SEM image of a hybrid In_2_O_3_–Pt NPs porous channel layer. (**d**) Response characteristic of the alcohol sensor toward ethanol in the concentration range of 95–952 ppb. (**e**) Schematic illustration of a wireless alcohol sensor attached to a steering wheel of an automobile. (**f**) Real-time ethanol sensing property of the wireless alcohol sensor attached to the steering wheel and transmitting data through Bluetooth communication. (**g**) Schematic illustration of the alcohol sensor integrated with an antenna on a smartwatch. (**h**) Ethanol sensing property of the battery-free wireless sensing module by monitoring resonant frequency shifts in the ethanol concentration range of 95–952 ppb. Reproduced with permission from Ref. [90] Copyright (2017), Elsevier. (**i**) Schematic illustrations of the synthesis of Cr_2_O_3_–SnO_2_ bilayer sensor. (**j**) Cross-sectional SEM image of SnO_2_ hollow sphere covered by Cr_2_O_3_ NPs. (**k**) Dynamic resistance transitions of the Cr_2_O_3_–SnO_2_ sensor toward ethylene in the concentration range of 0.1–2.5 ppm. Camera images of (**l**) IoT-based wireless sensor module and (**m**) real-time fruit freshness test. (**n**) Dynamic resistance transitions of three bananas with different levels of ripening using the sensing system. Reproduced with permission from Ref. [92] Copyright (2020), Wiley-VCH.

Gas sensor systems integrated with IoT-based wireless sensing modules can be employed as portable gas sensors for POCT [85,86]. The sensing data measured through a sensor can be transmitted to a mobile device for the real-time and on-site detection of various gas species [87,88,89]. Recently, the applicability of a POCT platform for ethanol detection has been demonstrated for adherence to safe driving requirements [90]. A flexible and transparent alcohol gas sensor was fabricated using an In_2_O_3_–Pt NP hybrid composite on a polyimide (PI) film (Figure 7a). Sensing electrodes were patterned using Ag NWs on a flexible PI after the deposition of In_2_O_3_–Pt NPs to detect ethanol (Figure 7b). The ethanol sensing layer of In_2_O_3_ was synthesized by the sol–gel technique to enhance its surface area, and Pt NPs were spray-coated onto the In_2_O_3_ sensing layer. The hybrid nanostructure of In_2_O_3_–Pt NPs was porous, which accelerated gas diffusion into the sensing layer (Figure 7c). 

The ethanol sensing properties of In_2_O_3_–Pt NPs were investigated by calculating the response (I_g_/I_a_), where I_a_ and I_g_ are the currents of the sensor in baseline dry air and in the presence of ethanol vapor, respectively (Figure 7d). The ethanol responses of In_2_O_3_–Pt NPs were 12.2 and 90.8 at concentrations of 95 and 952 ppb, respectively. Additionally, a 90-fold improvement in the response of the In_2_O_3_–Pt NP sensor was achieved in relation to that of the pristine In_2_O_3_ sensor toward 952 ppb ethanol. The drastic improvement in the response of the In_2_O_3_–Pt NP sensor was confirmed by XPS, wherein the intensity of the adsorbed oxygen species increased from 2% to 8% upon the addition of Pt NPs, which induces conductivity changes upon reaction with ethanol. Fast response and recovery times (1 and 2 s, respectively) were achieved with In_2_O_3_–Pt NPs at room temperature. In addition, consistent gas responses toward 95 ppb ethanol were achieved during 5000 bending cycles, thereby confirming reliable ethanol sensing performance of the In_2_O_3_-Pt NP sensor upon mechanical deformation.

A wireless ethanol sensing system was developed to transmit sensing results to a smartphone through Bluetooth communication. The flexible alcohol sensor was attached to the steering wheel of an automobile (Figure 7e), and the wireless sensing module detected ethanol vapor and evaluated the concentration of blood alcohol on the basis of ethanol vapor concentration (Figure 7f). Moreover, the In_2_O_3_–Pt NP sensor can be incorporated with antenna coils to eliminate the requirement of an external power source. A battery-free wireless ethanol sensing module was developed using an inner coil comprising a spiral pattern of Ag NWs for the antenna and an outer coil comprising the In_2_O_3_–Pt NP channel for the sensing layer (Figure 7g). The sensing response was measured by the reflection coefficient (S11), which indicates the power reflected from the transmitter to the antenna. The measured reflection values at a resonant frequency of 4.1 GHz decreased from −17 to −23 dB with increasing ethanol concentrations from 95 to 952 ppb (Figure 7h). The battery-free wireless sensing module can be attached to a smartwatch to monitor the blood alcohol concentration in real-time. 

In addition to their application of a wireless gas-sensing platform in POCT, such sensors are employed in IoT-based sensor systems for monitoring food quality in the agricultural industry [91,92,93]. For example, SnO_2_ hollow spheres were synthesized with a nanoscale Cr_2_O_3_ catalytic overlayer for improved selectivity toward ethylene, which is an important plant hormone used to determine the development and growth of climacteric fruits [92]. The overall fabrication process for the Cr_2_O_3_–SnO_2_ sensor is illustrated in Figure 7i. SnO_2_ hollow spheres were synthesized via one-pot ultrasonic spray pyrolysis. The precursors of tin (II) chloride dihydrate, citric acid monohydrate, and dilute hydrochloric acid solution (35.0–37.0%, HCl:DI water = 1:99 by vol%) were dissolved in DI water to obtain a spray solution. The Sn-containing precursor powder generated by the ultrasonic transducers was collected and converted to SnO_2_ hollow spheres by heat-treatment at 600 °C for 2 h. The SnO_2_ sensing layer was coated by the screen-printing method on an alumina substrate, and Cr_2_O_3_ catalytic overlayers were deposited on the sensing film through e-beam evaporation. The microstructure and surface morphology of the bilayered Cr_2_O_3_–SnO_2_ sensor were investigated by cross-sectional SEM, which indicated that the SnO_2_ hollow sphere was covered by Cr_2_O_3_ NPs with a thickness of 0.3 µm (Figure 7j). 

The gas-sensing performance of the bilayered Cr_2_O_3_–SnO_2_ sensor was evaluated toward ethylene in the concentration range of 0.1–2.5 ppm at 375 °C (Figure 7k). The Cr_2_O_3_–SnO_2_ bilayer sensor exhibited a significantly high response (R_air_/R_gas_ − 1 = 12.1) toward 2.5 ppm ethylene. The detection limit was 0.1 ppm with a corresponding response of 1.2. The Cr_2_O_3_–SnO_2_ bilayer sensor exhibited significantly improved selectivity toward ethylene against other interfering gases (e.g., trimethylamine, dimethylamine, ammonia, ethanol, formaldehyde, and carbon monoxide). The improved selectivity was mainly attributed to the decrease in response to interfering gases, which were converted to less-reactive species (e.g., CO_2_ and H_2_O) by the Cr_2_O_3_ catalytic layer.

The practical applicability of the Cr_2_O_3_–SnO_2_ sensor for monitoring fruit freshness to reflect the real-life storage of foods was demonstrated. An IoT-based wireless sensing module was developed with the Cr_2_O_3_–SnO_2_ sensor to monitor the freshness of bananas via ethylene detection (Figure 7l). The measured ethylene sensing data were transmitted to a smartphone in real-time (Figure 7m). The Cr_2_O_3_–SnO_2_ sensor distinguished the freshness of three bananas (under-ripened, ripened, and over-ripened) on the basis of resistance transitions resulting from an increase in ethylene concentrations as the bananas ripened (Figure 7n).

Based on recent developments in nanomaterials and IoT-based sensing systems, various emerging applications will be further explored, revealing the major advantages of nanostructured gas sensor systems in processes such as real-time analysis, rapid screening of multiple analytes, and wireless data transmission to mobile devices with improved sensitivity and selectivity, as summarized in Table 1.

## 3. Ion Sensors

The development of innovative ion sensor systems, including sensing materials, sensor substrates, and signal transduction techniques, enables real-time analysis through the rapid detection of analyte species, minimization of sensing platforms, and quantitative analysis of ion concentrations [96]. Advanced ion sensors can be integrated with IoT devices for developing portable and wearable sensing platforms [97]. Particularly, wearable sensing platforms have been developed for the analysis of biofluids, including sweat, considering their major advantages such as high efficiency for non-invasive healthcare monitoring and POCT [98,99].

Anion detection is gaining considerable attention in various fields, including healthcare, environmental monitoring, and biotechnology. For example, acetate (AcO^−^) is a metabolic switch that controls the rate of bacterial cell growth. In an abundant nutrient environment, bacterial cells such as *Escherichia coli* (*E. coli*) grow rapidly and excrete AcO^−^ [100,101,102]. The bacterial cells switch to a slower growth rate when their nutrients are depleted in the environment to enhance survival. The accumulation of AcO^−^ can inhibit cell growth and lower the productivity of recombinant proteins (e.g., synthetic insulin). Moreover, chloride (Cl^−^) in sweat is an important biomarker for the diagnosis of cystic fibrosis [103]. Increased Cl^−^ concentration in the range of 60–150 mM in sweat is generally observed in cystic fibrosis patients, whereas the normal Cl^−^ concentration range of a healthy individual is 10–40 mM [104].

Various receptors have been synthesized to detect anions, and their binding affinities toward specific anions have been evaluated. There has been particular interest in the design of receptor structures using dual-hydrogen bond donors such as urea, thiourea, deltamide, squaramide, and croconamide, considering their geometrical uniqueness for the binding of halides and Y-shaped oxoanions forming stable six- and eight-membered chelated structures, respectively [105,106]. Improvement in the anion-binding affinities of dual-hydrogen bond donors has been achieved by modulating *N*,*N*-substitutional functional groups. The anion-binding behavior of receptors comprising dual-hydrogen bond donors was characterized either by hydrogen bond interactions or deprotonation upon the injection of anions, depending on the acidity of the receptors. 

Various signal transduction techniques have been employed to understand the anion-binding behavior, such as those utilizing optical, magnetic, and electrochemical signals. Among the electrochemical signal transduction techniques, chemiresistive-type anion sensors have attracted considerable attention because of their ability for the real-time detection of anions with rapid screening and potential for integration with wireless sensing modules (Table 2). 

**Figure 8:**
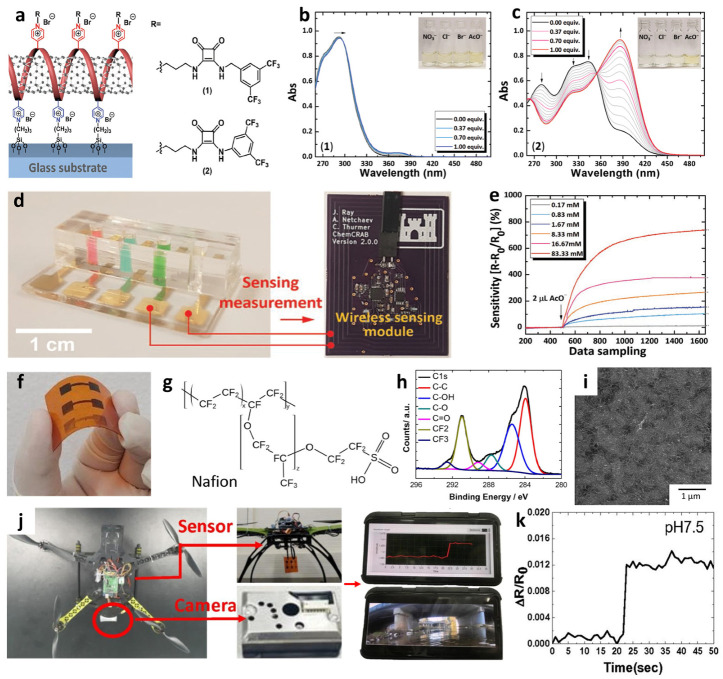
(**a**) Schematic illustration of an anion sensor with surface-anchored P4VP-SWCNT composite and anion selectors (1) and (2). UV-vis titrations of selector (**b**) (1) ([(**1**)] = 4.4 × 10^−5^ M) and (**c**) (2) ([(**2**)] = 4.5 × 10^−5^ M) upon the addition of up to 1 equivalent AcO^−^. (**d**) IoT-based anion-sensing platform composed of a sensor array and a wireless sensing module. (**e**) Real-time wireless detection of AcO^−^ in the concentration range of 0.17–83.33 mM. Reprinted with permission from Ref. [107] Copyright (2019), Wiley-VCH. (**f**) Chemical structure of a Nafion. (**g**) Camera image of screen-printed SWCNT-Nafion composite on a PI substrate. (**h**) XPS spectra and (**i**) SEM image of the SWCNT-Nafion composite film. (**j**) Real-time wireless pH sensing system by integrating the SWCNT-Nafion film sensor with a drone and transmitting the sensing data to a smartphone. (**k**) Real-time wireless pH monitoring of a river. Reproduced with permission from Ref. [112] Copyright (2019), Elsevier.

Recently, multiplexed chemiresistive anion sensors have been developed for the detection of AcO^−^ facilitating deprotonation of dual-hydrogen bond donors and electrical transduction using single-walled carbon nanotubes (SWCNTs) [107]. To fabricate the anion sensor, poly(4-vinylpyridine) (P4VP)-wrapped SWCNTs (P4VP-SWCNT) were patterned by spray-coating method, followed by the non-covalent functionalization of selectors composed of squaramide-based dual-hydrogen bond donors (Figure 8a). Specifically, a homogeneous dispersion of SWCNTs was prepared in *N*,*N*-Dimethylformamide (DMF) by dissolving P4VP and wrapping the SWCNTs. To prepare the sensing substrate, parallel Au electrodes were patterned on a glass substrate by depositing Au/Cr layers using a thermal evaporator. Subsequently, the glass substrate was treated with 3-bromopropyltrichlorosilane to form bromo alkyl chains on the surface. The mechanically stable P4VP-SWCNT composite was anchored on a surface-treated glass substrate by a quaternization reaction, wherein SWCNT-wrapped P4VP was covalently linked to the surface by the reaction between the pyridyl groups of P4VP and the bromo alkyl chains on the glass substrate. To induce selective anion-binding interactions, squaramide-based selectors were functionalized on the P4VP-SWCNT with different electron-withdrawing 3,5-bis(trifluoromethyl)benzyl (**1**) and 3,5-bis(trifluoromethyl)phenyl (**2**) groups (Figure 8a). The model structures of (**1**) and (**2**) were synthesized by *N*,*N*-substitution of squaramide with cationic moieties (e.g., pyridinium) and electron-withdrawing groups to systemically investigate the binding affinities toward various anions such as AcO^−^, Cl^−^, bromide (Br^−^), and nitrate (NO_3_^−^). 

The anion-binding properties of (**1**) and (**2**) were evaluated by UV-vis titrations upon the addition of AcO^−^ in dimethyl sulfoxide (Figure 8b–c). For the model selector (**1**), minor shifts in the absorption band at 292 nm were observed upon the addition of AcO^−^ up to 1 equivalent. A binding stoichiometry of 1:1 was confirmed by the Job curve, implying a hydrogen bond interaction between AcO^−^ and (**1**). For (**2**), an increased absorption band at 386 nm and decreased absorption bands at 280, 325, and 343 nm were observed upon the addition of up to 1 equivalent AcO^−^. The characteristic absorption spectra of (**2**) indicate the occurrence of hydrogen bond interactions between AcO^−^ and (**2**) with 1:1 binding stoichiometry at a low concentration (<1 equivalent) followed by the deprotonation of (**2**), resulting in the formation of a hydrogen-bond self-complex ((H(AcO)_2_)^−^) with 1:2 binding stoichiometry. The UV-vis titrations of (**1**) and (**2**) exhibited minor changes toward Cl^−^, Br^−^, and NO_3_^−^, which confirmed the occurrence of weak hydrogen bond interactions.

The anion-sensing properties of functional P4VP-SWCNTs with different electron-withdrawing groups, i.e., P4VP-(**1**)-SWCNT and P4VP-(**2**)-SWCNT, were evaluated for various anions such as AcO^−^, Cl^−^, Br^−^, and NO_3_^−^. A baseline solution of 10 μL acetonitrile was injected to establish the baseline resistance before the addition of the analyte solution. After stabilizing the sensor resistance, a 2 μL solution containing the target anion was injected to measure the resistance transitions. The sensor response was defined as the normalized resistance, i.e., (R–R_0_)/R_0_ (%), where R and R_0_ are the resistances upon the addition of the analyte solution and baseline solvent, respectively. The results revealed that P4VP-(**1**)-SWCNT exhibited a response of 7.34% toward AcO^−^ at 16.7 mM, followed by Cl^−^ > Br^−^ > NO_3_^−^. An approximately 16-fold higher response was achieved using P4VP-(**2**)-SWCNT with a response of 120.27% upon the addition of 16.7 mM AcO^−^. In terms of the selectivity of P4VP-(**2**)-SWCNT, the highest response was obtained with AcO^−^ followed by Br^−^ > Cl^−^ > NO_3_^−^.

Real-time wireless anion sensing was demonstrated using P4VP-(**2**)-SWCNT by integrating an anion sensor with a wireless sensor module (Figure 8d). The resistance changes were measured using the sensing module, and the sensing data were transmitted to a smartphone through near-field communication (NFC). Increasing response transitions were observed by increasing the AcO^−^ concentrations in the range of 0.17–83.33 mM (Figure 8e). The detection limit was 0.17 mM with a response of 12.39%. The resistance transitions upon the addition of AcO^−^ were mainly attributed to the internal charge transfer of (**2**) induced by the deprotonation of the squaramide. Increasing resistance transitions resulted from the negatively charged selector (**2**) after deprotonation, which traps hole carriers in the SWCNTs. The chemiresistive-sensing platform enables the real-time wireless anion detection of AcO^−^ by integration with an IoT sensing module.

Chemiresistive ion sensors using SWCNTs with different functional components can be employed for the detection of different ionic species such as proton (H^+^). The development of pH sensors by monitoring H^+^ concentrations is important for applications in healthcare systems and for water quality monitoring. For example, the normal pH of the sweat of a healthy person is 4.0–6.8, whereas an increased pH level of >9 is observed in patients with cystic fibrosis [108,109]. Additionally, pH values can indicate exercise intensity and dehydration levels [110]. In this regard, the pH level of individuals is closely related to their health conditions. In terms of monitoring pH in the environment, lowering the pH levels in seawater results in ocean acidification, threatening marine organisms that use calcium carbonate for their structural components [111]. Therefore, simple and portable pH measurement systems with wireless data transmission modules must be developed for the continuous monitoring of health and environmental conditions.

A wireless pH sensing system was demonstrated by facilitating a screen-printed SWCNT-Nafion nanocomposite on a flexible PI film [112]. The SWCNT was mixed with Nafion-117 at a concentration of 5%, which is the optimal condition for the screen-printing process. Nafion is composed of a hydrophobic backbone and hydrophilic side chains with sulfonic acid moieties, resulting in high proton conductivity (Figure 8f) [113,114]. The SWCNT-Nafion nanocomposite with a thickness of 40 μm was screen-printed on the PI substrate followed by heat-treatment at 100 °C for 10 min in the air (Figure 8g). A polydimethylsiloxane (PDMS) layer was attached to the top of the SWCNT-Nafion nanocomposite to protect the electrical contacts from pH buffer solutions.

The material properties and wireless pH sensing performance of the SWCNT-Nafion nanocomposite films were investigated. From the XPS spectra, two major peaks at 283.9 and 290.9 eV were assigned to the carbon–carbon interactions from C–C sp^2^ and the carbon–fluorine interactions from CF_2_, respectively (Figure 8h). These peaks are attributed to the binding of the Nafion chains with the SWCNTs. The surface morphology of the SWCNT-Nafion nanocomposite film was investigated by SEM (Figure 8i). Dense SWCNT networks were formed after coating by the screen-printing method with multiple electrical conducting paths. Real-time wireless pH sensing in a river was demonstrated by integrating the SWCNT-Nafion sensor with a drone (Figure 8j). The resistance transition was measured by immersing the SWCNT-Nafion sensor in a river and indicated a pH value of 7.5 (Figure 8k). The measured data were transmitted to a smartphone via wireless communication, enabling water quality monitoring from remote locations. The sensing mechanism revealed that the formation of negatively charged OH^−^ interacts with C–H bonds from the SWCNT-Nafion nanocomposite, wherein the majority of hole carriers in the SWCNTs are immobilized in a basic environment, leading to an increase in resistivity. On the other hand, H^+^ produced in an acidic environment binds to C–O bonds in the SWCNT-Nafion composite, which results in decreased resistivity because hole carriers are donated back to the SWCNTs. 

**Table 2:** Recent development of chemiresistive ion sensors for IoT applications.

Material	Response Definition	Response	Detection Limit	Testing Ambient	Target Ions	Applications	Ref.
SWCNT-P4VP-Squaramide	ΔR/R_0_ (%)	120.27% @ 16.7 mM	1.7 mM	Acetonitrile	CH_3_COO^−^	IoT sensor	[107]
SWCNT-Nafion	ΔR/R_0_	~0.2 @ pH 12	-	H_2_O	H^+^	IoT sensor	[112]
SWCNT-P4VP-Thiourea	ΔR/R_0_ (%)	101.9% @ 16.7 mM	0.17 mM	Acetonitrile	CH_3_COO^−^	-	[115]
SWCNT-P4VP-Croconamide	ΔR/R_0_ (%)	140.91% @ 83.33 mM	0.17 mM	Acetonitrile	CH_3_COO^−^	-	[116]

As a different type of electrochemical ion sensor, potentiometric sensors that facilitate potential differences across selective ion-sensing membranes have been developed for the detection of cationic species integrated with a wireless sensing module for POCT applications. Potentiometric sensors are advantageous because of the simplicity of operation, low power consumption, and their potential for miniaturization [117,118,119]. Recently, wearable-type potentiometric ion sensors (WPISs) integrated with IoT sensing modules have gained significant attention for their applicability toward the real-time monitoring of ion concentration changes in body fluids for healthcare management, sports performance monitoring, and physiological analysis [120,121]. Additionally, they can be employed for the diagnosis of critical nervous disorders and heart failure by monitoring metabolic indicators such as Na^+^, K^+^, and pH [122,123]. The recent development of WPISs in South Korea is summarized in Table 3 for the detection of Na^+^, K^+^, and H^+^.

**Figure 9:**
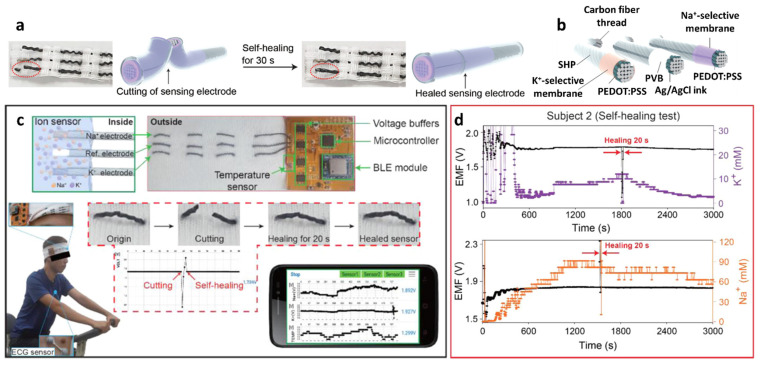
(**a**) Schematic illustrations and photographic images of the self-healing process of PCSC-coated CFT electrodes. (**b**) Schematic illustration of the PCSC-coated K^+^ ISE, reference electrode, and Na^+^ ISE. (**c**) Application of the self-healing ISE to the flexible ion-sensing platform, communicating with a wireless sensing device. Data acquisition from a headband-shaped sensor. (**d**) Simultaneous data acquisition of Na^+^ and K^+^ from sweat using the sensor platform during exercise. Cutting and healing processes occurred during signal collection. Reproduced with permission from Ref. [133] Copyright (2019), American Chemical Society.

The development of a mechanically robust potentiometric wearable sensing platform is important, considering that the sensor is subjected to constant mechanical stress during human natural activities such as walking, running, and stretching [124,125,126,127]. To address this issue, fibrous wearable sensors with self-healing polymers (SHPs) have been proposed for the autonomous repair of damage [128,129,130,131,132]. Yoon et al. developed a wearable sweat sensor for the detection of Na^+^/K^+^ using SHPs, i.e., poly (1,4-cyclohexanedimethanol succinate-co-citrate) (PCSC)-coated carbon fiber thread (CFT) electrodes [133]. The self-healing polymer can be restored with >97.0% healing efficiency within 30 s at room temperature (Figure 9a).

To fabricate a wearable sweat monitoring sensor, a self-healable PCSC polymer was first synthesized by the addition of citric acid (CA; 9.51 g, 49.5 mmol), succinic acid (SA; 6.82 g, 57.8 mmol), and 1,4-cyclohexanedimethanol with 74 mol% trans-isomer (19.0 g, 132 mmol) by the esterification process. The mixture in the reactant-containing dry vessel was stirred for 105 min under a nitrogen atmosphere at 160 °C and then poured onto a Teflon sheet. The mechanical properties of the PCSC were as follows: Young’s modulus, E = 340 MPa; ultimate tensile strength, σ = 2.8 MPa; elongation at break, ε = 350%; and toughness, U = 7.7 MJ m^−3^. The self-healing property of the PCSC was nearly instantaneous as the toughness was recovered by 85% after 30 s and 92% after 60 s of cutting the PCSC film. The self-healing property of the PCSC is attributed to moderately cross-linked oligomers containing terminal carboxylic acid and alcohol groups as self-healing motifs by forming a reversible pseudo-network via an intermolecular hydrogen bonding [134,135].

To prepare the Na^+^/K^+^ ion-selective electrode (ISE), the CFT surface was electrochemically deposited with poly(3,4-ethylenedioxythiophene) polystyrene sulfonate, which acts as a solid contact transducer (Figure 9b). Ion-selective membranes (ISMs) were prepared by dipping the CFT in a cocktail solution for membrane coating. The cocktail solution was prepared by mixing 1:2 *w*/*w* of poly(vinyl chloride) (PVC) and a dioctyl sebacate (DOS) polymer matrix, lipophilic ionophores (i.e., Na ionophore X and valinomycin for Na^+^ and K^+^ sensing, respectively), and ion exchangers (e.g., sodium tetrakis [3,5-bis(trifluoromethyl)-phenyl]borate (Na-TFPB) for Na^+^ and potassium tetrakis(4-chlorophenyl)borate for K^+^) in 1 mL of tetrahydrofuran (THF). The Ag/AgCl reference electrode was formed by coating the CFT with an Ag/AgCl ink. To prevent unwanted potential drift during potentiometric sensing, the as-obtained Ag/AgCl-coated CFT was immersed in a MeOH solution containing 50 mg of NaCl and 78 mg of BUTVAR B-98 (PVB) followed by drying at room temperature. The high concentration of NaCl in the PVB polymer electrolyte helps the Ag/AgCl half-cell to maintain the reference potential by providing Cl^−^ [136]. Finally, the ISEs and reference electrodes were coated with PCSC (10 vol%) dissolved in a mixture of chloroform (10 mL) and dimethylacetamide (10 mL).

The sensing properties of the PCSC–CFT-based ISE were evaluated for Na^+^ and K^+^ in the concentration range of 0.1–100 mM under various physical conditions such as normal, bent, and crumpled states. Under the normal condition, the PCSC–CFT Na^+^/K^+^ ISE showed linear Nernstian slopes of 60.7 ± 1.5 mV log[Na^+^]^−1^ (i.e., mV per decade) (R^2^ = 0.99) and 54.8 ± 0.6 mV log[K^+^]^−1^ (R^2^ = 0.99) (*n* = 5). Rapid electromotive force (EMF) signal detection was achieved over 10–20 s with high stability at 16–60 °C. In addition, the sensors exhibited stable signal detection even under severe mechanical bending and crumpling conditions. Moreover, the K^+^ sensor exhibited similar responses of 55.0 and 54.9 mV log[K^+^]^−1^ for the bent and crumpled states, respectively. For the detection of Na^+^, minor differences in the response values were obtained for the bent (59.4 mV log[Na^+^]^−1^) and crumpled states (59.3 mV log[Na^+^]^−1^).

The PCSC–CFT-based ISE was integrated with a wireless flexible printed circuit board (FPCB) for real-time sweat monitoring. The FPCB is composed of a PCSC–CFT-based sensor, a temperature sensor, interface circuits, a microcontroller, a Bluetooth low energy system, and a Li-ion battery (3.7 V). For sweat monitoring, the on-body sensing test was performed using a headband sweat sensor fabricated by knitting PCSC–CFT on a fabric. Healthy volunteers exercised on a stationary bike for 50 min at room temperature while wearing the headband PCSC–CFT-based sweat sensor (Figure 9c). The signal profiles of the on-body sweat electrolytes revealed that Na^+^ and K^+^ concentrations increased rapidly and then stabilized with a small decrease. (Figure 9d) [26,122,137]. To evaluate the sensor performance, an on-body test using a commercial electrochemical analyzer was conducted. The obtained K^+^ and Na^+^ signals from the FPCB-integrated PCSC–CFT sensor during exercise were consistent with the sensing results of the electrochemical analyzer. The self-healing performance of the PCSC-coated CFT electrodes was demonstrated by cutting and reattaching them during stationary exercise. When the sensors were cut into two pieces during stationary exercise, the signal fluctuation was observed as a result of the disconnection of the electrochemical cell. After 20 s of healing time, the sensing signal was restored to the original state. This work demonstrated the applicability of the headband-type sweat monitoring sensor for the detection of Na^+^ and K^+^ with mechanical robustness, biocompatibility, and low energy consumption.

**Figure 10:**
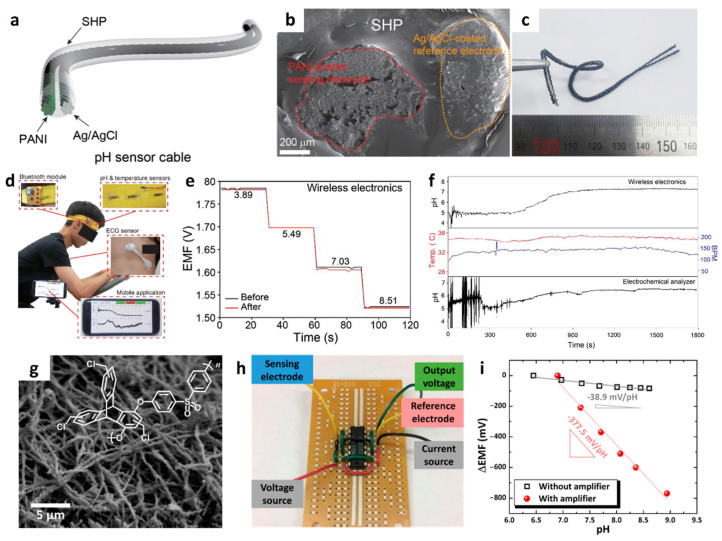
(**a**) Schematic illustration, (**b**) cross-section SEM image, and (**c**) camera image of a cable-type potentiometric pH sensor. (**d**) Real-time sweat monitoring by the detection of pH levels using a wearable headband-type wireless sensor platform communicating with a smartphone. (**e**) Potentiometric EMF titrations of the pH sensor before and after the on-body test. (**f**) Real-time pH monitoring of body fluids during exercise. Reproduced with permission from Ref. [138] Copyright (2020), Elsevier. (**g**) SEM image of WO_3_ NFs and chemical structure of Cl-TPES. (**h**) Camera image of a MOSFET-based differential amplifier. (**i**) Potentiometric pH responses with and without the integration of a differential amplifier. Reproduced with permission from Ref. [141] Copyright (2019), American Chemical Society.

The mechanically robust self-healable polymer can be further utilized for the detection of different ionic species such as H^+^ for monitoring body pH levels. Wearable body fluid pH sensors were developed using PCSC–CFT electrodes incorporated with pH-sensitive polyaniline (PANI) to facilitate redox equilibrium between H_3_O^+^ and PANI phase transitions [138,139]. The PANI pH sensing layer was prepared by the electrochemical deposition of aniline monomers onto the CFT surface (~1 × 10 mm^2^) by cyclic voltammetry (CV). The deposition proceeded with CV for 30 cycles over a potential range of –0.1 to +0.8 V at 0.5 M H_2_SO_4_ containing 0.25 M aniline monomer. The reference electrode was prepared by coating the carbon fiber surface with an Ag/AgCl ink. The Ag/AgCl-coated CFT was protected by PVB containing NaCl and dried at room temperature. The obtained PANI-coated CFT working electrode and Ag/AgCl-coated reference electrode was coated with PCSC dissolved in a mixture of chloroform and dimethylacetamide through the dip-coating method. Finally, a cable-type flexible and self-healing pH sensor was fabricated by weaving PCSC-coated electrodes (Figure 10a–c). The cross-sectional SEM image revealed that each electrode was composed of carbon fibers with a diameter of ~10 μm. The overall diameter of the cable-type pH sensor was less than 3 mm.

The sensing performance of the flexible pH sensor cables was evaluated by immersing them in a buffer solution with a pH of 3.89–10.09. The flexible pH sensor cable exhibited a linear Nernstian slope of 58.28 mV/pH in the range of 3.89–10.09 (0.86% relative standard deviation (RSD), R^2^ = 0.9979, *n* = 5), 58.9 mV/pH (RSD 0.84% and R^2^ = 0.9981) at pH 4.0–7.0, 57.5 mV/pH (RSD 0.85% and R^2^ = 0.9931) at pH 6.0–8.0, and 58.9 mV/pH (RSD 0.86% and R^2^ = 0.9964) at pH 4.0–8.0. The response time of the pH sensor was 5 s when the pH level increased from 4.73 to 8.02. Highly selective pH sensing properties were obtained with minor potential changes against other interfering cations such as Na^+^, K^+^, NH_4_^+^, Ca^2+^, and Mg^2+^ with selectivity coefficients of <1, calculated by the separate solution method [140]. The self-healing property of the sensor demonstrated that the damaged sensor was healed within 5.4 s and its sensing signal was completely restored (healing efficiency > 97.8%) after it was cut into two pieces at room temperature. 

A flexible pH sensor cable was integrated with an FPCB wireless module to measure the pH levels in body fluids. The on-body test demonstrated the practical application of the wearable pH sensor. A headband-type wearable pH sensor was prepared by knitting the FPCB-integrated pH cable with a fabric (Figure 10d). The pH levels measured by monitoring the EMF changes were collected using a wearable pH sensor during stationary exercise. Consistent EMF changes were confirmed by measuring the pH levels using both a wearable sensor and a reference electrochemical analyzer, which ensured reliability in sensing measurements. Additionally, the change in the calibration curves was negligible before and after the on-body test (Figure 10e). These results indicate the stability of pH sensor cables for wearable applications. During the exercise, a sufficient volume of sweat was collected after 5 min, and a pH value of 7.34 was measured from the body fluids using the wearable pH sensor (Figure 10f). 

Potentiometric pH sensors can be applied in environmental monitoring, such as ocean acidity, to measure the pH levels of seawater [141]. For the pH sensing layer, a 1D fiber composite was prepared by mixing WO_3_ NFs and a binding polymer. WO_3_ exhibits high pH selectivity upon reaction with H^+^ by forming hydrogenated tungsten bronzes (H_x_WO_3_), as described below [142,143]:

(3) $${\mathrm{WO}}_{3}+{\mathrm{xH}}^{+}+{\mathrm{xe}}^{-}\;↔\;H_{x}{\mathrm{WO}}_{3}\;(0<x<1)$$

For the improved pH-sensitive layer with a large surface area and high porosity, 1D WO_3_ NFs were synthesized by electrospinning process followed by high-temperature calcination. Specifically, an aqueous composite solution was prepared by dissolving PVP and the W precursor [(NH_4_)_6_H_2_W_12_O_40_·xH_2_O] in DI water. Electrospinning was performed by ejecting the solution into a collector under a high voltage applied to the solution. As-spun PVP/W fibers with a diameter < 5 μm were calcinated at 800 °C to remove the polymeric components and oxidize the W precursor. As a result, continuous inorganic WO_3_ NFs were obtained with a reduced diameter of approximately 500 nm and multiple mesoscale (2–50 μm) pores on the surface (Figure 10g). Subsequently, chloromethylated triptycene poly(ether sulfone) (CI-TPES) as a permeable binder was homogeneously mixed with WO_3_ NFs (WO_3_ NFs/CI-TPES) to improve the mechanical stability.

Moreover, the potentiometric pH sensing properties of WO_3_ NFs/CI-TPES were investigated by measuring the EMF signals with respect to an Ag/AgCl reference electrode. WO_3_ NFs/CI-TPES exhibited a Nernstian slope of −38.9 mV/pH (R = 0.9274, pH range of 6.45–8.75), which is 50.3% higher than that of pristine WO_3_ NFs (−25.6 mV/pH, R = 0.9833, pH range of 6.45–8.61). To overcome the Nernstian limit, i.e., (59.16/z) mV/log a_i_, signal amplification was proposed by integrating a metal-oxide field-effect transistor (MOSFET) as a differential amplifier (Figure 10h). A dramatically improved pH response was achieved with the differential amplifier, as a greater voltage output was produced with a linear Nernstian slope of −377.5 mV/pH (R = 0.9847) in the pH range of 6.90–8.94. The Nernstian slope after the integration of the differential amplifier exhibited an improvement of an order of magnitude compared to the pH sensor without a differential amplifier and 6.4-fold higher than the Nernstian limit (Figure 10i). For potential applications in monitoring ocean acidity, pH sensing characterization was performed using artificial seawater containing interfering ions such as Na^+^, Mg^2+^, Ca^2+^, Cl^−^, and SO_4_^2−^. The pH level was adjusted by adding NaHCO_3_ as a source of HCO_3_^−^ to mimic ocean acidification. During pH titrations from 8.08 (current pH level of the ocean) to 7.9, the WO_3_ NFs/CI-TPES sensor showed small EMF changes less than 3 mV without the differential amplifier. In contrast, the amplifier-enhanced pH sensor produced a significantly high output of up to 175 mV with an improved signal-to-noise ratio. Further integration of an IoT sensing module with a WO_3_ NFs/CI-TPES-based potentiometric sensor and a differential amplifier can allow high-resolution pH monitoring for the simultaneous analysis of ocean acidification at multiple locations.

**Figure 11:**
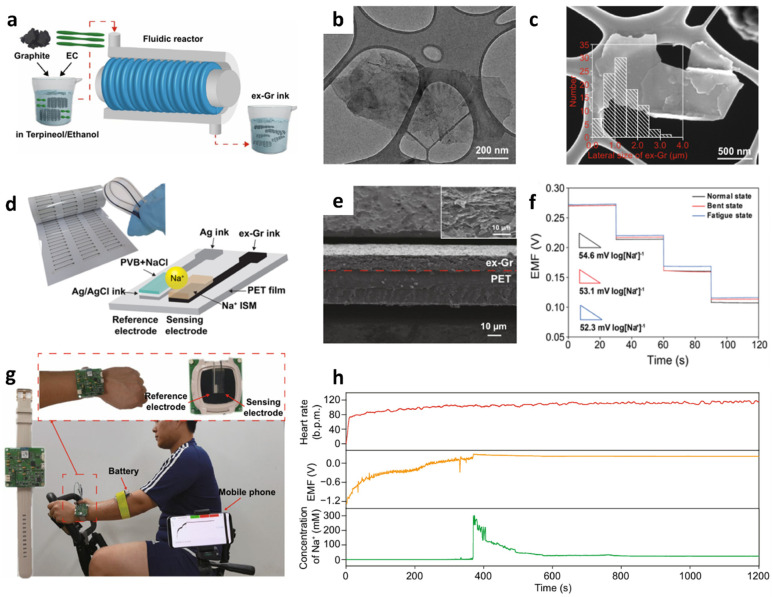
(**a**) Schematic illustration of ex-Gr ink preparation through fluid dynamics-induced exfoliation process. (**b**) TEM and (**c**) SEM images of the ex-Gr sheets with the histogram for the lateral size distribution in the inset. (**d**) Photographic image and schematic structure of a screen-printed Na^+^ sensor. (**e**) Cross-sectional SEM image of the printed ex-Gr conductor with the high-resolution SEM image at the interface between printed ex-Gr and PET substrate in the inset. (**f**) Potentiometric EMF transitions of the sensor in the Na^+^ concentration range of 10^−1^–10^−4^ M under mechanically normal, bent, and fatigue states. (**g**) Camera images of wristwatch-type wearable Na^+^ sensors and real-time wireless data acquisition with a smartphone during stationary exercise. (**h**) Simultaneous data acquisition of a subject’s heart rate, sensor responses, and converted Na^+^ concentration during exercise. Reproduced with permission from Ref. [147] Copyright (2021), Springer Nature.

In terms of sensing materials for the development of wearable sensors, mechanical flexibility with high electrical conductivity is important. Low-dimensional nanomaterials such as 1D CNTs, 2D graphene, and 2D MXenes are gaining much attention owing to their high conductivity, large surface area, flexibility, and durability [144,145,146]. Recently, a screen-printed wearable Na^+^ sensor based on a conductive graphene ink transducer with high electrical conductivity was integrated with a wristwatch-type device [147]. To prepare graphene ink, exfoliated graphene (ex-Gr) was synthesized by the fluid dynamics-induced exfoliation and mixing process, resulting in a defect-free ex-Gr with high yield (Figure 11a). A 9:1 *w*/*w* mixture of graphite and ethylene carbonate (EC) dispersed in terpineol/ethanol (5:5 *v*/*v*) was loaded in a fluidic reactor and processed at 2000 rpm for 2 h. Graphene was exfoliated from the graphite using the shear field of the Taylor vortex, and EC was used to suspend the exfoliated graphene flakes in an organic solvent and to enhance the adhesion between the ex-Gr and printing substrates. An ex-Gr ink was obtained after centrifugation followed by drying at 225 °C for 24 h under vacuum to remove terpineol and ethyl cellulose. TEM and SEM analysis revealed that thin-layered ex-Gr flakes were synthesized with an average lateral size of 1.10 ± 0.84 μm (Figure 11b–c). The thickness of ex-Gr was 1.2 ± 0.8 nm as confirmed by atomic force microscopy indicating multilayered ex-Gr nanosheets.

A flexible Na^+^-selective electrode sensor was fabricated using screen-printed ex-Gr as the transducer layer (Figure 11d). Electrical circuit electrodes were patterned on a flexible polyethylene terephthalate (PET) film using a commercial screen printer and a stainless-steel mask. The cross-sectional SEM image showed good adhesion at the interface of the printed ex-Gr and PET substrate (Figure 11e). Conductive inks composed of ex-Gr and Ag/AgCl were used to form working and reference electrodes, respectively, followed by an annealing process at 200 °C for 120 min to improve the conductivity (σ = 1.49 × 10^4^ S m^−1^). A Na^+^-selective electrode sensor was fabricated by coating the printed ex-Gr with a Na^+^ ISM cocktail. The membrane cocktail was prepared by dissolving PVC (31.5 wt%), DOS (67 wt%), Na ionophore X (1 wt%), and Na-TFPB (0.5 wt%) in THF. The printed Ag/AgCl reference electrode was coated with a mixture of 50 mg NaCl and 78 mg PVB for signal stabilization.

The potentiometric Na^+^ sensing performance was evaluated by measuring the EMF between the Na^+^-ISM and Ag/AgCl reference electrodes in the concentration range of 10^−1^–10^−4^ using NaCl solution as a Na^+^ source. The ex-Gr-based sensor exhibited a Nernstian slope of 54.0 ± 0.65 mV log[Na^+^]^−1^ (*n* = 5). Moreover, the response time was 3.6 s, which was measured by successively increasing the Na^+^ concentration from 1 to 100 mM. The detection limit of the sensor was calculated as 14.8 μM. The Na^+^ sensing performance was mainly attributed to the large surface area and the formation of the electrical double-layer capacitance of the ex-Gr transducer. The mechanical and electrical stabilities of the printed ex-Gr-based Na^+^ sensor were investigated under 1400 cycles of bending and fatigue tests (Figure 11f). Negligible differences in sensitivity were observed between the normal (54.0 ± 0.65 mV log[Na^+^]^−1^) and bent states (53.1 mV log[Na^+^]^−1^). In addition, the sensor showed minor changes in response (52.3 mV log[Na^+^]^−1^) after the fatigue test, demonstrating the stable sensing performance of ex-Gr-based Na^+^-ISE under mechanical stresses.

A wristwatch-type wearable Na^+^ sensor was fabricated by integrating a potentiometric Na^+^-selective sensor and a PCB with a wireless module for application in an on-body test (Figure 11g). Before data acquisition, the Na^+^ sensor was calibrated using a commercial electrochemical analyzer. An on-body test was conducted during stationary biking at room temperature. After a sufficient amount of sweat collection (~370 s), the potentiometric signal was stabilized confirming that the Na^+^ concentration at 24 mM was measured via a smartphone, which is the normal physiological Na^+^ concentration (Figure 11h).

**Figure 12:**
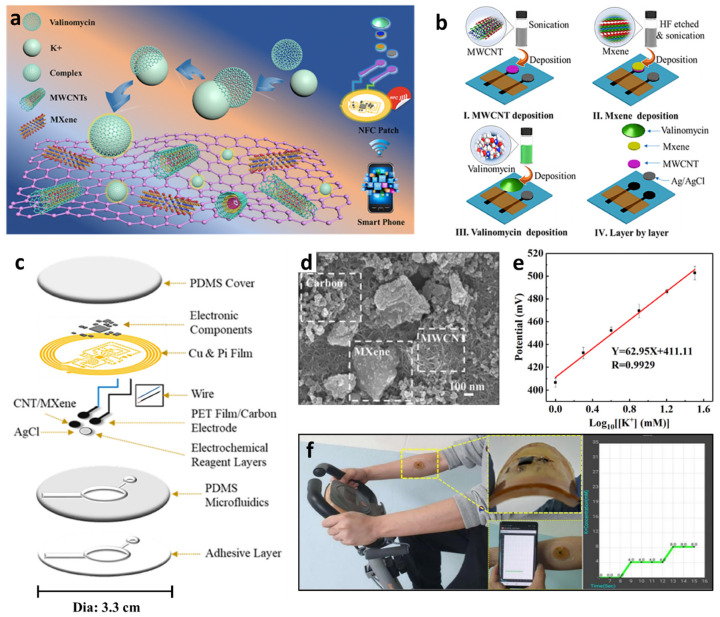
(**a**) Schematic illustration of the battery-free electrochemical sensing NFC patch based on MWCNTs–MXene hybrid K^+^ sensing layer. (**b**) Schematic illustration of the fabrication of a screen-printed K^+^-selective sensor and the reference electrode. (**c**) Schematic illustration of the exploded view of a patch-type integrated sensor system. (**d**) SEM image of MWCNTs/MXene–Ti_3_C_2_T_x_ hybrid networks on a carbon electrode. (**e**) K^+^ response characterization with the Nernstian slope in the K^+^ concentration range of 1–32 mM. (**f**) Real-time on-body sweat monitoring with an NFC patch-type K^+^ sensor via a smartphone during stationary exercise. Reproduced with permission from Ref. [148] Copyright (2021), Elsevier.

A wearable perspiration sensor for K^+^ detection was developed using a hybrid multidimensional carbon-based material combined with 2D MXene [148]. MXene–Ti_3_C_2_T_x_ 2D nanosheets are promising materials owing to their outstanding electrical conductivity and large surface area. However, their discrete distribution and agglomeration pose challenges in their application in functional sensing materials [149,150,151,152]. To address these issues, the hybrid sensing materials of 2D MXene–Ti_3_C_2_T_x_ and 1D multi-walled carbon nanotube (MWCNT) networks have been proposed as electrical transducers. Figure 12a shows a conceptual illustration of the MWCNT/MXene–Ti_3_C_2_T_x_-based K^+^ flexible sensors with an NFC wireless sensing module. MXene–Ti_3_C_2_T_x_ 2D nanosheets were prepared by etching and exfoliating the MAX-phase Ti_3_C_2_T_x_ using minimally intensive layer delamination synthesis method with 9 M HCl/12 M LiF etchant [153]. Ti_3_C_2_T_x_ powder was added to the etchant and stirred for 24 h at room temperature. MXene–Ti_3_C_2_T_x_ nanosheets were obtained after several centrifuging, washing, sonication, and drying processes. In parallel, an MWCNT suspension was prepared by dispersing 24 mg of MWCNTs in 12 mL of DMF for sensor fabrication.

Flexible K^+^ sensor electrodes were fabricated on a PET substrate using a screen-printing technique (Figure 12b). Carbon paste was drop-casted on a PET with a masking layer, followed by a layer-by-layer modification of the electrode using 5 μL of MWCNT suspension and 5 μL of MXene-Ti_3_C_2_T_x_ suspension. Finally, a K^+^-selective PVC–DOS membrane containing valinomycin was drop-casted. A reference electrode was fabricated by drop-casting Ag/AgCl paste onto the carbon electrode. Screen-printed flexible reference and working electrodes were integrated with a radio frequency electromagnetic energy harvester for an NFC wireless patch with a battery-free operation. Figure 12c shows schematic images of the integrated sensor electrodes, NFC electronics, and microfluidic system. An ultra-thin (thickness = 32 μm) flexible circuit was patterned on the PI substrate, and the circuit pattern was fabricated by photolithography and wet etching of a copper foil-coated PI film on a silicon wafer. A microfluidic system based on 3D printed PDMS mold was adopted to collect sweat and mitigate surface contamination. A light and small patch sensor system (diameter = 3.3) was prepared to transmit data in real-time to a smartphone with wireless communication. 

By the layer-by-layer coating, 1D MWCNTs were intercalated to 2D MXene–Ti_3_C_2_T_x_ nanosheets to provide a “bridging effect” as shown in the SEM image (Figure 12d) [154]. As a result, the hybrid structure of MWCNT/MXene–Ti_3_C_2_T_x_ exhibited excellent electrical conductivity and a large surface area. Compared to pristine MWCNTs and MXene–Ti_3_C_2_T_x_, hybrid MWCNTs/MXene–Ti_3_C_2_T_x_ showed a larger CV area, indicating improved double-layer capacitance and electrocatalytic activity (5 mM Fe(CN)_6_^3−/4−^, 0.1 M KCl, 50 mV s^−1^ scan rate). Electrochemical impedance spectroscopy (EIS) analysis revealed that the hybrid MWCNTs/MXene had a lower R_ct_ (~203 Ω) compared to bare carbon (~3.8 kΩ), carbon/MWCNT (~3.4 kΩ), and carbon/MXene (~611 Ω), confirming the high electrical conductivity of the hybrid structure. 

Potentiometric K^+^ sensing performance was evaluated with data acquisition using a smartphone upon the addition of K^+^ ions at concentrations of 1–32 mM. Step-like increases in the potential values were obtained by increasing the K^+^ concentration with a fast response time of 2 s. The Nernstian slope was 62.95 mV/dec (R = 0.9929), which is close to the theoretical value of 59.16 mV/dec (Figure 12e). Excellent K^+^ selectivity was achieved with no sensing signals for other body fluid ions such as Ca^2+^, Na^+^, and Zn^2+^. Moreover, real-time on-body monitoring of K^+^ concentration was demonstrated by integrating K^+^ ISE with an NFC patch-type sensor (Figure 12f). The sensor detected K^+^ concentration after a certain amount of sweat collection in the microfluidic channel. The battery-free patch-type wearable sensor based on the hybrid MWCNTs/MXene–Ti_3_C_2_T_x_ material exhibited selective K^+^ sensing capability, demonstrating its potential application in human perspiration analysis for healthcare and physiological studies.

**Table 3:** Recent developments of potentiometric ion sensors for IoT applications.

Material	Response	Dynamic Range	Testing Ambient	Target Ions	Response/Recovery Time	Applications	Ref.
PCSC-coated CFT	60.7 ± 1.5 mV log[Na^+^]^−1^ 54.8 ± 0.6 mV log[K^+^]^−1^	10^−1^–10^−4^ M	Body fluid (sweat)	Na^+^, K^+^	10–20 s	IoT wearable sensor	[133]
PCSC-coated CFT	58.28 mV/pH	pH 3.89–10.09	Body fluid (sweat)	H^+^	5 s	IoT wearable sensor	[138]
WO_3_ NFs/ CI-TPES	−377.5 mV/pH (With differential amp.)	pH 6.90–8.94	Artificial seawater	H^+^	-	Ocean acidification monitoring	[141]
Defect-free exfoliated graphene	54.0 mV log [Na^+^]^−1^	10^−1^–10^−4^ M	Body fluid (sweat)	Na^+^	3.6 s	IoT wearable sensor	[147]
MWCNTs– MXene (Ti_3_C_2_T_X_)	63 mV log [K^+^]^−1^	1–32 mM	Body fluid (sweat)	K^+^	2 s	IoT wearable sensor	[148]

## 4. Biosensors

Innovations in the development of biosensors integrated with biological elements and signal transducers enable the development of a new generation of sensor systems for POCT with rapid and precise detection of biological signals. Flexible and wearable sensor systems integrated with IoT sensing platforms have been developed to detect various biomolecules in the human body, such as glucose, lactate, uric acid, and bacteria (e.g., pathogenic *Escherichia coli*) [155,156,157]. In particular, novel glucose sensors are gaining considerable attention worldwide because of their applicability in the non-invasive diagnosis of diabetes mellitus through continuous glucose monitoring (CGM) [158,159,160]. Early diagnosis of diabetes by monitoring glucose levels is of significant importance, considering that the total global diabetes population is expected to increase by over 50% in 2045 compared to the estimated number of diabetes patients worldwide in 2017 [161]. Patients with diabetes experience uncontrolled blood glucose levels as a result of chronic hyperglycemia, causing various diabetic complications such as blindness, nerve damage, cardiovascular disease, and kidney failure [162]. Therefore, adequate medical treatment and prevention of diabetes should be achieved by continuous real-time monitoring of blood glucose levels.

Electrochemical glucose sensors have been widely utilized by facilitating enzymatic reactions for CGM [159]. In particular, the glucose oxidase (GO_x_) enzyme has been commonly employed owing to several advantages such as high specificity toward glucose, stability over various pH levels, and temperature changes [163]. The basic principle of glucose sensors using GO_x_ is based on the oxidation of glucose via an enzymatic reaction that produces gluconic acid and hydrogen peroxide (H_2_O_2_) in the presence of oxygen, as shown in the following reaction [156]:

(4) $$\mathrm{Glucose}\;+{\;H}_{2}O\;+{\;O}_{2}\;\overset{\;\;\mathrm{GOx}\;\;}{\to }\;\mathrm{Gluconic}\;\mathrm{acid}\;+{\;H}_{2}O_{2}$$

Toward the development of enzymatic glucose sensors using GO_x_, three generations have been established depending on the mechanism of charge transfer to the sensing electrode [156,158]. The first generation of glucose sensors indicates the amount of glucose oxidation as a result of an enzymatic reaction, which is monitored by measuring either oxygen consumption or H_2_O_2_ production. The first-generation glucose sensors exhibit major advantages such as simplicity and potential for miniaturization; thus, they can be applied for in vitro and in vivo clinical trials [164]. However, a high overpotential for the detection of H_2_O_2_ causes side reactions of electroactive species, resulting in low selectivity toward the target analyte. The second generation of glucose sensors involves the use of redox mediators with GO_x_, wherein the mediators interact directly with enzymes and an electrical current signal is generated upon the addition of glucose as a result of the redox reaction of the mediator. For the third generation of GO_x_-based glucose sensors, electron transfer occurs by direct interaction between the enzyme and the electrode without incorporating mediators. Generally, engineered enzymes are utilized to combine the electrode and GO_x_ through structural modification, resulting in direct electron exchange. For example, GO_x_ enzymes are coupled with porous polymeric membrane electrodes or nanostructured carbon nanotube electrodes to facilitate electron transfer [165,166,167,168].

**Figure 13:**
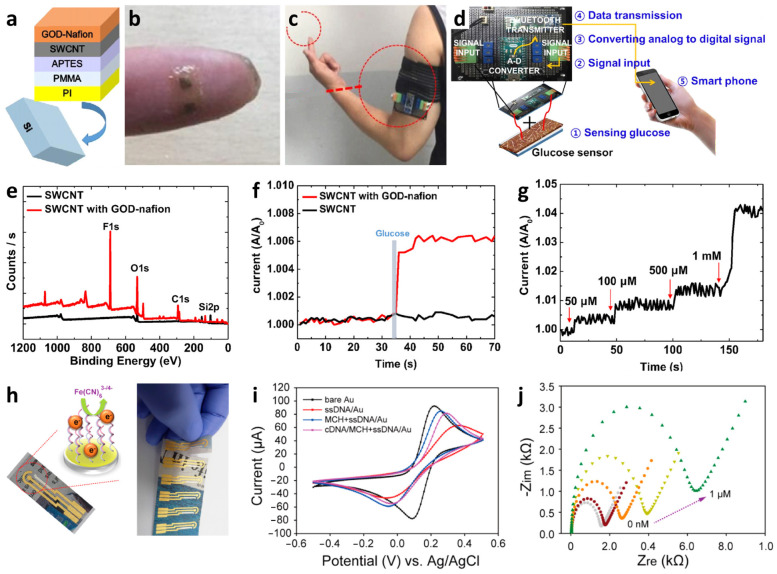
(**a**) Schematic illustration of a wearable SWCNT-based glucose sensor fabricated by an all-solution process. Camera images of (**b**) a wearable SWCNT-based glucose sensor on a finger and (**c**) integrated with a wearable sensing module. (**d**) Wearable glucose sensor system integrated with an IoT-based sensing module to transmit glucose-sensing data to a mobile device. (**e**) XPS spectra analysis to confirm the functionalization of the GO_x_-Nafion composite on an SWCNT film. (**f**) Real-time glucose-sensing property of pristine SWCNT and GO_x_-Nafion-composite-functionalized SWCNT upon exposure to 50 μM of glucose. (**g**) Real-time response changes of GO_x_-Nafion-composite-functionalized SWCNT upon successive addition of glucose ranging from 50 μM to 1 mM. Reprinted with permission from Ref. [169] Copyright (2019), Elsevier. (**h**) Disposable-paper-based electrochemical sensors after coating parylene C (P-paper) and electrodes for the detection of foodborne pathogens (i.e., cDNA of *E*. *coli* O157:H7). (**i**) CV curve to investigate the step-by-step assembly process of ssDNA probe immobilization, MCH blocking monolayer formation, and cDNA hybridization. (**j**) Nyquist plots of the paper-based sensor upon exposure to different concentrations of target cDNA. Reprinted with permission from Ref. [172] Copyright (2016), American Chemical Society.

Kang et al. demonstrated a wearable glucose-sensing system using GO_x_-Nafion-composite-functionalized SWCNTs, which can be categorized as a third-generation GO_x_-based glucose sensor [169]. The multilayered structure of GOx-Nafion-composite-functionalized SWCNTs on a flexible substrate was achieved by an all-solution process (Figure 13a). Specifically, a thin layer of PI with a thickness of 30 μm was coated on a Si wafer as a substrate, followed by the deposition of 1 μm of poly(methyl methacrylate) (PMMA) by spin-coating. Subsequently, the substrate was immersed in a 3-(aminopropyl)triethoxysilane (APTES) solution to form amine groups on the surface. A dispersion of SWCNTs (length ranging from 100 nm to 4 μm; diameter of 1.2–1.7 nm) in 1,2-dichlorobenzene (1 mg/100 mL) was deposited through spray-coating onto the APTES-modified PMMA/PI/Si substrate having a thickness of 3–7 nm followed by annealing at 150 °C for 30 min. Thus, dense SWCNT networks were formed as a result of Coulombic interactions between the SWCNTs and the amine groups from the APTES layer. Finally, a composite solution of GO_x_ and Nafion-117 was covered on the SWCNT networks through spin-coating. The composite layers were detached from the Si substrate resulting in a flexible and wearable glucose sensor, which can be directly attached to the human skin to monitor glucose concentration using a smartphone in real-time (Figure 13a,b). A wearable glucose sensor system was established by integrating a small glucose sensor (1 cm × 1 cm in dimension) and an armband-type sensing module to transmit the sensing signal to a smartphone (Figure 13c,d). 

Material characterization and glucose-sensing performance of the fabricated sensor were investigated (Figure 13e–g). The XPS survey analysis confirmed the surface functionalization of the SWCNT networks with the GO_x_-Nafion composites, wherein peaks related to fluoride, oxygen, and sulfur were observed as a result of surface functionalization (Figure 13e). On the other hand, the XPS survey spectrum of pristine SWCNTs exhibited no relevant peaks of GO_x_-nafion composites. Real-time wireless glucose-sensing properties of the wearable SWCNT-based glucose sensor systems were evaluated by monitoring the response transitions defined by A/A_0_, where A_0_ and A are the initial current before exposure to glucose and measured current after the injection of glucose, respectively. The results revealed that there is a sudden increase in the current of the SWCNTs functionalized with GO_x_-Nafion composites upon exposure to 50 μM glucose, whereas there were no changes in the current signal from the pristine SWCNTs (Figure 13f). The current response transitions upon successive injection of glucose were investigated in the range of 50 μM–1 mM (Figure 13g). Increasing current responses for the SWCNT-based glucose sensor functionalized with the GO_x_-Nafion composite were observed with continuously increasing glucose concentrations. The glucose-sensing mechanism is based on the conductance of SWCNT networks affected by the enzymatic oxidation of glucose by GO_x_. The fundamental principle of glucose oxidation can be explained by the formation of oxidized flavin adenine dinucleotide (FAD) as a sub-unit of the GO_x_ enzyme from the reduced form of FAD (i.e., FADH_2_), while catalytically oxidizing glucose [170]. The increasing current upon the injection of glucose is mainly attributed to the direct electron transfer to the SWCNT networks during the oxidation of FAD [171]. 

Various flexible biosensor platforms for the detection of biological analytes have been developed on flexible substrates and applied for the point-of-care (POC) diagnosis [172,173]. For example, a flexible biosensor composed of a multilayered GO_x_/gold/MoS_2_/gold nanofilm on a PI substrate was demonstrated to be applicable for glucose detection [173]. The multilayer structure was fabricated by sputtering gold on the PI film and subsequently depositing MoS_2_ NPs through the spin-coating method. The gold sputtering process was performed again to form a gold/MoS_2_/gold nanofilm on a PI substrate with the dimension of 2.5 × 20 mm. To induce glucose-sensing properties, GO_x_ was immobilized on a gold surface assisted by a chemical linker. The amperometric glucose-sensing result of the GO_x_/gold/MoS_2_/gold nanofilm revealed that a rapid increase in the current signal was obtained upon the addition of glucose with a limit of detection of 10 nM. The improved glucose-sensing response was mainly attributed to efficient electron transfer by the MoS_2_ NPs during catalytic glucose oxidation. 

The development of a biosensing platform composed of a paper substrate is advantageous considering its major advantages such as simplicity of fabrication, low cost, and large-scale production of sensor devices [174]. Flexible biosensors with scalable and cost-effective strategies have been demonstrated using a disposable paper substrate. For example, a waste newspaper was employed as a sensor substrate for the detection of pathogenic *Escherichia coli* O157:H7 (*E. coli* O157:H7) using an electrochemical measurement technique [172]. The disposable paper was coated with parylene C (P-paper) to enhance its mechanical properties and increase its hydrophobicity while maintaining its porous nature [175]. After patterning the sensing electrodes on the P-paper, a self-assembled capture probe monolayer, i.e., single-strand probe DNA (ssDNA), was immobilized on a sensing electrode, followed by the formation of a 6-mercapto-1-hexanol (MCH) monolayer to block nonspecific binding to the bare gold electrode. Subsequently, a hybridization reaction was conducted by injecting synthetic cDNA or denatured amplicons of *E. coli* O157:H7 as a model foodborne pathogen (Figure 13h). CV and EIS were performed to investigate the step-by-step assembly process and the target cDNA detection capability. The CV characteristics of the aqueous 5 mM Fe(CN)_6_^3−/4−^ electrolyte solution revealed that the peak current was significantly decreased with an increase in peak-to-peak separation (Δ*E*_P_) from 110 mV for the bare Au electrode to 310 mV after the immobilization of the ssDNA probe and blocking with the MCH monolayer (Figure 13i). Further decreased peak current and increased Δ*E*_P_ (330 mV) were achieved after hybridization with the target cDNA. EIS further confirmed the cDNA detection capability at different concentrations using P-paper-based sensors in the presence of Fe(CN)_6_^3−/4−^ as an indicator (Figure 13j). Nyquist plots revealed gradually increased charge transfer resistance (*R*_ct_) upon increasing the target cDNA concentration. This result indicates binding between the ssDNA probe and cDNA, which results in a negatively charged surface leading to the attenuation of electron transfer. 

**Figure 14:**
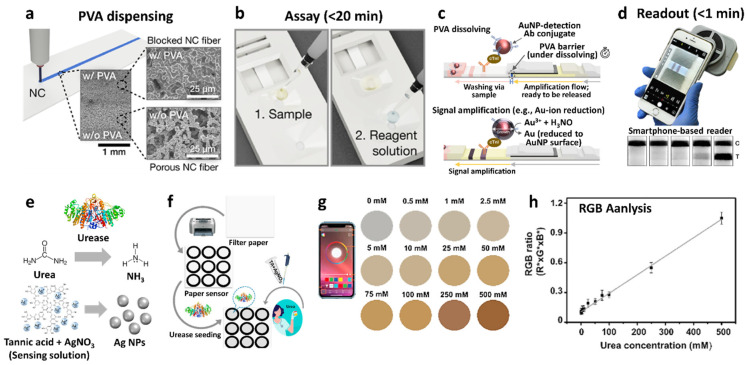
(**a**) Dispensing of PVA on a nitrocellulose (NC) membrane for the LFA test platform with SEM images. (**b**) LFA test process by the injection of sample and reagent solutions. (**c**) Schematic illustration of colorimetric Au-ion amplification facilitating automated reaction/fluid switching mechanism by dissolving the PVA barrier. (**d**) Colorimetric signal readout using a smartphone. Reprinted with permission from Ref. [182] Copyright (2020), American Chemical Society. (**e**) Schematic illustration of the mechanism of the colorimetric urea biosensors, generation of NH_3_ by an enzymatic reaction between urea and urease, and pH-responsive reduction of Ag^+^ ions to Ag NPs by tannic acid. (**f**) Schematic illustration of the fabrication of a colorimetric urea biosensor. (**g**) Analysis of colorimetric urea biosensor based on the calculated RGB ratio at different urea concentrations. (**h**) Smartphone-assisted RGB ratio measurement in the urea concentration range of 0–500 mM. Reprinted with permission from Ref. [183] Copyright (2021), Elsevier.

As a different type of transduction mechanism, colorimetric sensing mechanism has been utilized to detect biomolecules and viruses because of their simple visual readout and their capability to rapidly screen multiple analytes with high portability [176,177,178,179,180,181]. Several studies have been conducted to fabricate colorimetric biosensors using a paper substrate paired with a smartphone-based reader for application in POCT [182,183,184,185,186]. A paper/soluble polymer hybrid-based biosensing platform was developed for the diagnosis of myocardial infarction by detecting human cardiac troponin I (cTnI) as a standard biomarker [182]. Among the various biosensing platforms for POCT applications, lateral flow assays (LFAs) are the most widely used because of their major advantages such as affordability, simplified device architecture, user-friendliness, ability for rapid detection, robustness, and long shelf life (~2 years) under ambient conditions [186,187,188,189]. However, because of the relatively low sensitivity of conventional LFAs, they cannot be effectively applied for biomarker detection in the concentration as low as sub-ng/mL. To overcome this limitation, a paper-based LFA with signal amplification (i.e., signal-amplification-based LFA) was proposed to facilitate biochemical reactions to further enhance sensitivity and promote quantitative analysis [190,191]. To this end, low-cost and mass-produced batch-type test strips were prepared to analyze cTnI, incorporated with a smartphone-based reader for high-performance POCT. The paper/polyvinyl alcohol (PVA) hybrid was patterned by dispensing the PVA solution on nitrocellulose (NC) membrane, which plays a key role in programmable fluid control and automated fluid switching (Figure 14a). The test proceeds with an assay followed by a signal readout using a smartphone. The assay was performed by the injection of a sample solution containing a cTnI biomarker to induce immunoreaction and a reagent solution to activate the amplification reaction within 20 min (Figure 14b). The mixture fluid injected through a reagent pad gradually dissolved the patterned PVA barrier, resulting in fluid switching from the sample fluid to the amplification fluid (Figure 14c). As a result, the test platform realized automated signal amplification reactions at the test and control lines. Various amplification techniques have been demonstrated such as Au-ion amplification, wherein gold ions (Au^3+^) were reduced to Au NPs in the presence of a reducing agent (H_3_NO), thereby generating amplified colorimetric signal changes. The enhanced colorimetric signal was proportional to the amount of reduced Au NPs, which were formed after the immunocomplex reaction. The intensity of color changes was measured using a smartphone reader after 20 min of the assay (Figure 14d). The result revealed excellent analytical sensitivity with a detection limit of 0.92 pg/mL cTnI and a coefficient of variation of <10% in serum or plasma samples comparable to those of commercially available standard analyzers, thereby demonstrating its potential application in POCT systems. 

Paper-based colorimetric biosensors facilitating smartphone-assisted analysis have been used for the detection of other biological samples. For example, urea is a biomolecule that is one of the products of the metabolism in the human kidneys and liver, providing pathophysiological information on renal and hepatic disorders. To develop a POC bioassay for urea detection, a colorimetric biosensor was proposed to facilitate the hydrolysis of urea, and a sensing solution composed of tannic acid and AgNO_3_ was subsequently added, which resulted in the reduction of Ag ions to form Ag NPs (Figure 14e) [183]. A circular pattern of a hydrophilic sampling zone was laser-printed on a paper sheet, wherein a sampling solution containing urea and urease as well as a sensing solution were deposited (Figure 14f). Urea was first hydrolyzed by urease, inducing an enzymatic reaction while forming NH_3_ and CO_2_ as products. The production of NH_3_ changed the solution pH, inducing the reduction of Ag^+^ ions into Ag NPs by tannic acid and generating a yellow color. The smartphone RGB software was used to analyze color changes upon the production of Ag NPs at different urea concentrations (Figure 14g). The smartphone-assisted POC colorimetric sensing was demonstrated by RGB ratio calculation based on the intensity variations with respect to urea concentrations (Figure 14h). The detection of urea in human urine samples was conducted without dilution, and a linear correlation was achieved with respect to the urea concentration (R^2^ = 0.993) with a detection limit of 0.58 mM in the detection range of 0–500 mM. The results demonstrated that the smartphone-assisted colorimetric biosensors can be used in POCT for the detection of urea and monitoring of renal or hepatic disorders. 

Recently, IoT-based biosensor platforms have gained considerable attention for the continuous monitoring of human health and the early diagnosis of certain diseases. One characteristic feature of the platform is the interconnection between a biosensor and a mobile device assisted by a sensing module through the internet, enabling wireless data transmission to a mobile device and data accumulation in the cloud system [192]. For the IoT-based biosensor platform, a biological interaction in a biosensor produces an electrical signal upon the injection of an analyte, and this signal can be displayed on a smartphone screen. Thus, such sensors can be employed in POC diagnosis. To this end, novel biosensors must be developed for promoting research of innovative materials and strategies for device fabrication. 

**Figure 15:**
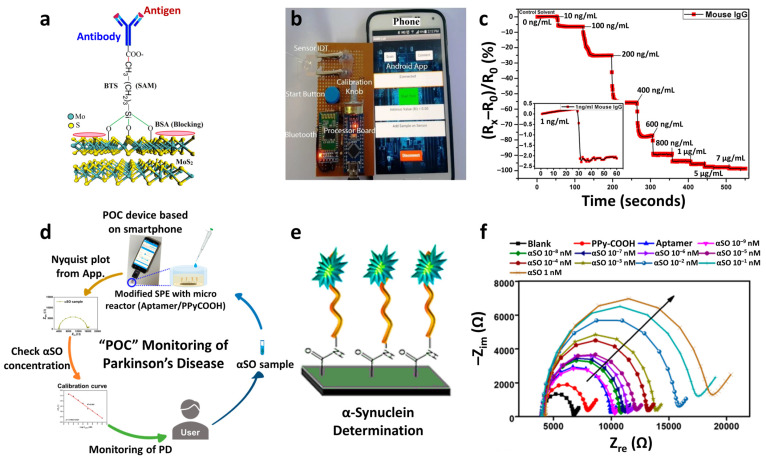
(**a**) Antibody–antigen complex formation on the MoS_2_ active layer. (**b**) Real-time monitoring of electrical response from the sensor for the detection of human PSA. (**c**) Step-like response transitions upon the increasing concentration of IgG with the limit of detection of 1 ng/mL in the inset. Reprinted with permission from Ref. [193] Copyright (2017), Nature Publishing Group. (**d**) Schematic illustration of the POC monitoring of PD using a smartphone. (**e**) Fabrication of an electrochemical biosensor for the detection of α-SOs by using a conductive polymer immobilized with an aptamer. (**f**) Nyquist plots of the biosensor in the presence of α-SOs in the concentration range of 10^−9^–1 nM. Reprinted with permission from Ref. [199] Copyright (2020), American Chemical Society.

Immunosensors with compact analytical devices have been developed for the quantitative detection of multiple bioreagents facilitated by antibody–antigen complex formation [193]. Immunosensors can transduce immunologic reactions between an antigen and an antibody into an electrical signal as measured by EIS. Various 2D nanosheets including TMDs are emerging electrochemical sensing layers because of their scalable bandgaps, which can customize their physical properties and optimize their electrical signal transduction properties [194,195]. Hence, highly porous 2D MoS_2_ sheets have been employed as active sensing layers on a glass substrate patterned with electrodes. Multilayered MoS_2_ was prepared by the mechanical exfoliation technique, wherein probe sonication was performed to exfoliate bulk MoS_2_ powder dispersed in *N*-methyl-2-pyrrolidone. Subsequently, the unexfoliated bulk MoS_2_ flakes were separated by centrifugation resulting in a clear MoS_2_ solution. To induce immunoreaction, multiple antibodies were physically immobilized on the MoS_2_ active layers, and a specific antigen such as mouse immunoglobulin G (IgG) was detected using the sensor (Figure 15a). POC diagnosis was demonstrated for the real-time detection of IgG by using a smartphone (Figure 15b). The sensing system was calibrated with standard solutions containing IgG at known concentrations. Subsequently, quantitative detection was performed with an unknown solution to evaluate sensitivity (%) and IgG antigen concentration, which were displayed on the smartphone screen. The electrochemical impedance measurement revealed the detection limit at 1 ng/mL IgG in the detection range of 1 ng/mL–9 μg/mL (Figure 15c). In addition, reproducible POC diagnosis was confirmed by measuring stable IgG sensing responses with an average error of ±5.8% in the IgG concentration range of 0–600 ng/mL. The use of a specific antibody as a bioreceptor can provide high specificity and reliable responses to immunosensors. However, the use of antibodies as bioreceptors exhibits several disadvantages such as time consumption in the development of specific antibodies, high cost of production, and short shelf life requiring continuous storage at low temperature [196]. 

Unlike antibodies serving as bioreceptors, aptamers are synthetic receptors possessing inherent advantages over antibodies, including relatively fast development time, low manufacturing cost, large-scale synthesis process, long shelf life, and selectivity toward target molecules [197,198]. Therefore, aptamer-based immunosensors have been integrated with IoT sensing platforms for the diagnosis of diseases [199]. A POC platform was developed by incorporating a conductive polymer with an aptamer for the early-stage diagnosis of Parkinson’s disease (PD) [199]. The platform composed of an electrochemical biosensor can measure electrical impedance signals in the presence of varied biomarker concentrations, and the analysis result can be monitored using a smartphone (Figure 15d). In terms of biomarkers for the early detection of PD, a lipophilic phosphoprotein α-synuclein (α-Syn) was found in the cerebrospinal fluid (CSF), which was considered a distinguished biomarker for PD [200]. The ability to detect α-Syn oligomers (α-SOs) can be exploited to evaluate future cognitive decline because picomolar concentrations of α-SOs correspond to the range of α-SO levels found in the CSF of PD patients [201]. An electrochemical biosensor was fabricated for the detection of α-SOs by employing a conductive polymer, i.e., pyrrole-2-carboxylic acid (PPy-COOH), which was deposited onto a screen-printed electrode by the electropolymerization process (Figure 15e). Subsequently, aptamers containing –NH_2_ groups were covalently immobilized in PPy-COOH through amide bond formation in order to allow the direct detection of α-SOs. The polymerization and immobilization steps were confirmed by EIS, wherein Nyquist plots revealed increased charge transfer resistances, i.e., semicircles, after each step (Figure 15f). EIS was conducted using a smartphone integrated with a portable potentiostat, and the measurement results were displayed on a smartphone screen. The results revealed that charge transfer resistance (R_et_) increased with increasing α-SO concentration, which is mainly attributed to the binding of α-SOs with the aptamer, preventing charge transfer between Fe(CN)_6_^3−/4−^ ions and the electrochemical probe. The calibration curve showed a linear relationship between the transitions in R_et_ (i.e., ΔR_et_) and the logarithmic concentration of α-SOs (i.e., −log C_α-SO_) with high reproducibility (*n* = 5) and linearity (R^2^ = 0.993). The EIS results confirmed the detection limit of 1 × 10^−^^3^ fM, which is suitable for the detection of α-SOs in saliva. The study demonstrated the applicability of a portable POCT platform for the early diagnosis of PD using a conductive polymer as a charge transducer immobilized with an aptamer by the detection of α-SOs at sub-femtomolar concentrations. 

Recently, the rapid on-site detection of viruses has gained considerable attention owing to the COVID-19 pandemic [202,203,204]. COVID-19 is a respiratory infectious disease caused by severe acute respiratory syndrome coronavirus 2 (SARS-CoV-2) with high infection and mortality rates [205,206]. Currently, the real-time reverse transcription-polymerase chain reaction (RT-PCR) is conducted in clinical laboratories for the detection of SARS-CoV-2 and is found to have high precision. However, a relatively long analysis time of at least 3 h and the need for professional experts with sophisticated analysis procedures limit the rapid screening of samples in large quantities [207]. Hence, the development of a new type of biosensor with a POCT platform is imperative for the detection of viral species to prevent the spread of SARS-CoV-2 by isolating patients [208,209,210,211,212]. 

**Figure 16:**
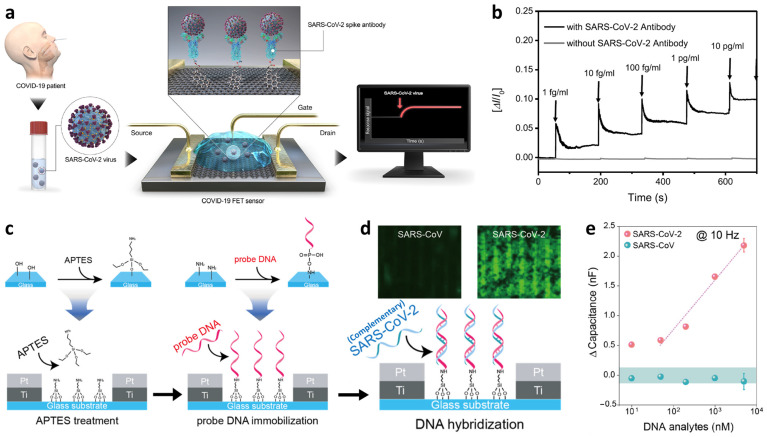
(**a**) Schematic illustration of the operating procedure of the FET sensor for the detection of SARS-CoV-2 in human nasopharyngeal swab specimens. (**b**) Real-time response of the COVID-19 FET sensor upon the injection of SARS-CoV-2 antigen protein in PBS. Reprinted with permission from Ref. [211] Copyright (2020), American Chemical Society. (**c**) Schematic illustrations of the probe DNA immobilization process using a surface modifier (APTES) and (**d**) hybridization reaction of analyte DNA with probe DNA for SARS-CoV-2 cDNA detection as confirmed by a fluorescent signal. (**e**) Capacitive response transitions (ΔC) of biosensors toward SARS-CoV and SARS-CoV-2 at concentration ranging from 10 nM to 5 μM. Reprinted with permission from Ref. [212] Copyright (2021), Elsevier.

Moreover, electrochemical biosensors have been developed for the rapid detection of COVID-19 causative virus (i.e., SARS-CoV-2) in human nasopharyngeal swab specimens, revealing their effective real-time and selective detection capabilities (Figure 16a) [211]. In that study, a field-effect transistor (FET)-based COVID-19 sensor was fabricated using graphene as a signal transduction layer functionalized with a SARS-CoV-2 spike antibody. Graphene layers synthesized on a copper foil were transferred onto a SiO_2_/Si substrate by the wet-transfer method to fabricate FET-based COVID-19 sensors. The dimension of the graphene-loaded FET sensor was 100 × 100 μm^2^ (L × W). To induce the selective detection of SARS-CoV-2, the SARS-CoV-2 spike antibody was immobilized on a graphene layer assisted by a coupling agent (e.g., 1-pyrenebutyric acid N-hydroxysuccinimide ester). The sensing characterization was performed by measuring the current between the source and drain upon the injection of the SARS-CoV-2 virus. The normalized current signal is defined as the response, (ΔI/I_0_) = (I − I_0_)/I_0_, where I_0_ and I are the initial and detected currents after the injection of the virus sample, respectively. The real-time sensing characterization using the graphene-based FET after antibody immobilization exhibited distinctive signal changes with a detection limit of 1 fg/mL in phosphate-buffered saline (PBS; pH 7.4) (Figure 16b). In contrast, pristine-graphene-based FET devices without antibody immobilization showed negligible response changes upon the injection of the SARS-CoV-2 spike protein. The specific binding property of the COVID-19 FET sensor was confirmed by the selective detection of the SARS-CoV-2 spike protein, whereas there was no response signal toward middle east respiratory syndrome coronavirus (MERS-CoV) spike proteins. To demonstrate the clinical applicability of the COVID-19 FET sensor, SARS-CoV-2 from nasopharyngeal swabs dispersed in a universal transport medium was utilized. The results revealed clear discrimination in the sensing responses between the samples from healthy subjects and those from COVID-19 patients. Thus, the COVID-19 FET sensors are applicable for POCT. 

Biosensors that promote cost-effective and real-time detection of SARS-CoV-2 have also been developed by facilitating capacitance signal changes upon the hybridization of analyte DNA with probe DNA [212]. The operational mechanism involves the immobilization of probe DNA using specific mRNA sequences in the SARS-CoV-2 gene on a glass substrate patterned with interdigitated sensing electrodes (Pt/Ti) (Figure 16c). To immobilize probe DNA on a glass substrate, surface modification using APTES was performed to form amine groups as linker molecules. Subsequently, probe DNA was covalently immobilized by reactions between the amine groups on the surface of the glass substrate and the phosphate group from probe DNA. The surface modification and immobilization steps were confirmed by measuring the capacitance-frequency properties. Increasing capacitance was observed in the low-frequency range (≤10 Hz) as a result of increased dielectric constants after functionalization with APTES (*ε* = 3.57) and the immobilization of probe DNA (*ε* = ~8) [213,214]. Probe DNA can induce hybridization with complementary SARS-CoV-2 cDNA, wherein double-stranded DNA (dsDNA) is formed through hydrogen bonds (Figure 16d). The formation of dsDNA was confirmed by a fluorescent signal, which revealed a strong, green-colored image after hybridization. The capacitance transitions were measured upon the hybridization induced by the injection of complementary SARS-CoV-2 target DNA at various concentrations in the range of 10 nM–5 μM (Figure 16e). The capacitive response was 0.843 nF/nM (red curve) after the hybridization of SARS-CoV-2 cDNA with a detection limit of 10 nM, whereas there was an invariant response toward non-complementary SARS-CoV cDNA. The proposed biosensors exhibited high sensitivity (ΔC = ~2 nF) and selectivity toward SARS-CoV-2 cDNA, which can be applied in the POCT platform for the rapid and cost-effective diagnosis of COVID-19.

## 5. Conclusions and Future Perspectives

The recent research progress in the development of chemical sensors in South Korea was comprehensively reviewed. Particularly, the development of nanomaterials and IoT sensor platforms are highlighted for applications in POCT and diagnosis. We classified three different types of chemical sensors based on the target chemical species: (i) gas sensors, (ii) ion sensors, and (iii) biosensors. For the development of gas sensors, multidimensional nanostructures, such as 1D fibers and 2D nanosheets, were developed on a flexible substrate. The flexible gas sensors were integrated with an IoT-based wireless sensing module to transmit the data to a smartphone, which allows the real-time and on-site detection of various environmental gases such as NO_2_ and H_2_S. For the development of ion sensors, chemiresistive, and potentiometric sensors were introduced for the detection of anions and cations. A multiplexed chemiresistive sensor array was developed by patterning SWCNTs with synthetic selectors, which was integrated with an NFC sensing module for the wireless detection of AcO^−^. Furthermore, various wearable potentiometric sensor systems such as patches and headbands have been developed with ISMs containing ionophores for the detection of Na^+^ and K^+^. Wearable potentiometric sensors were demonstrated for the analysis of body fluids during exercise for healthcare applications. Finally, novel biosensors have been demonstrated for the rapid screening of biomolecules and virus species. Electrochemical and colorimetric sensors were discussed for the detection of analytes facilitating enzymatic reactions and immunoassays. Moreover, mobile devices such as smartphones have been used to analyze color changes or electrical signal transitions of sensors upon interaction with biomolecules. 

There are remaining challenges and issues in nanomaterial-based sensing platforms, such as stability of nanomaterials, rapid on-site detection of analytes, sensor reliability, low power consumption, portability, and usability of sensing platforms. Therefore, further development of novel nanomaterials with improved sensing properties toward target analytes is desirable. In addition, user-friendly mobile sensing platforms can be further integrated with chemical sensors for continuous monitoring of personal health conditions and remote diagnosis. The demand for novel and effective IoT-based chemical sensors for POCT will continuously increase, considering the importance of personal healthcare and environmental monitoring. 

## Figures and Tables

**Table 1:** Recent development of gas sensors using multidimensional nanocomposites for IoT gas sensor and POCT applications.

Material	Response Definition	Response	Detection Limit	Testing Ambient	Target Gas	Response/Recovery Time	Applications	Ref.
PdO@Co_3_O_4_– SWCNT	(R_air_–R_gas_)/R_air_ (%)	44.11% @ 20 ppm	1 ppm	Air	NO_2_	-	Wearable sensor	[15]
Pt-nRGO fiber	(R_gas_–R_air_)/R_air_ (%)	3.53% @ 66.4% RH	-	Air	H_2_O	-	POCT	[46]
RGO fiber	(R_gas_–R_air_)/R_air_ (%)	0.39% @ 20 ppm	0.814 ppm	Dry air	NO_2_	108 s/ 72 s	Wearable sensor	[51]
ORGO	(R_0_–R)/R_0_ × 100%	2.69% @ 20 ppm	-	Ambient air	NO_2_	-	Wearable sensor, POCT	[52]
WO_3_ NRs-RGO composite fiber	(R_gas_–R_air_)/R_air_ (%)	9.67% @ 5 ppm	-	Dry air	NO_2_	180 s/ 432 s	Wearable sensor	[57]
PtPd–WO_3_	R_air_/R_gas_	97.5 @ 1 ppm	1.07 ppb	Humid air	Acetone	4.2 s/ 204 s	POCT	[61]
WS_2_@MTCNFs	(R_gas_–R_air_)/R_air_ (%)	15% @ 1 ppm	10 ppb	Dry air	NO_2_	5.7 min/ 3.7 min	IoT gas sensor	[63]
Pd@Ag HNWs	ΔR/R_0_ (%)	0.89% @ 900 ppm	100 ppm	Dry air	H_2_	120 s/ 102 s	Wearable sensor	[65]
ORGO	(R_air_–R_gas_)/R_air_ (%)	0.238% @ 20 ppm	1 ppm	Dry air	H_2_S	-	POCT	[75]
Pt_ZnO/PRGO	(R_air_–R_gas_)/R_air_ (%)	43.23% @ 5 ppm	0.1 ppm	Dry air	NO_2_	8.8 min/ 11.7 min	Wearable sensor	[80]
Optically punched RuO_2_	(R_gas_–R_air_)/R_air_ (%)	1.124% @ 20 ppm	-	Dry air	NO_2_	-	Wearable sensor	[82]
In_2_O_3_/Pt	I_g_/I_a_	90.8 @ 952 ppb	5.14 ppb	Dry air	Ethanol	1 s/ 2 s	IoT gas sensor, POCT	[90]
Cr_2_O_3_–SnO_2_	(R_air_–R_gas_)/R_air_	12.1 @ 2.5 ppm	24 ppb	Dry air	Ethylene	10 s/ 70 s	IoT gas sensor	[92]
Fluorinated graphene oxide (CFGO)	ΔR/R_0_ (%)	121% @ 500 ppm	6 ppb	Dry air	NH_3_	-	IoT gas sensor	[94]
Al-Doped ZnO Nanofiber	R_gas_/R_air_	11 @ 0.5 ppm	0.2 ppm	Dry air	NO_2_	23 s/ 40 s	IoT gas sensor	[95]

## Data Availability

Not applicable.
